# Supplementary material for: Chemically stable fluorescent proteins for advanced microscopy
Source: Nat Methods. 2022 Nov 7;19(12):1612–21. doi: 10.1038/s41592-022-01660-7 (PMC9718679; doi:10.1038/s41592-022-01660-7)
Supplement: Supplementary file 1 — Supplementary Data, Discussion, Note, Methods, References, Figures 1–8, Tables 1–3 and Videos 1 and 2. [file 41592_2022_1660_MOESM1_ESM.pdf]

nature methods

Article

https://doi.org/10.1038/s41592-022-01660-7

Chemically stable fluorescent proteins for advanced microscopy

In the format provided by the
authors and unedited

Chemically stable fluorescent proteins for advanced microscopy

Benjamin C. Campbell, Maria G. Paez-Segala, Loren L. Looger, Gregory A. Petsko, Ce Feng Liu

Supplementary Information

Supplementary Tables & Figures are located at the bottom of the document.

Supplementary Data **3**
- Equilibrium unfolding 3
- Fluorescent proteins with a reduced genetic code 3
- NaOH resistance and development of mhYFP 3
- Crystal structures 4
- Salt bridges on the hyperfolder protein surfaces 4

Supplementary Discussion **5**
- Structure-activity relationships in hfYFP, mhYFP, and FOLD6 5
- Additional observations related to chromophore maturation 6
- Mechanisms for maturation enhancement by the I167T mutation 6
- Hydrophobic packing in the hfYFP chromophore environment 8
- Discovery of the S147R mutation 8
- A third water molecule in the chromophore proton wire 9
- Slow denaturation in NaOH 9

Supplementary Note **10**
- Library development 10
- Development of cysteine-free fluorescent proteins 10
- Role of V68L in Tm and maturation rate 12
- Inspiration for the YFP templates 12
- Thr167 reversion to Ile167 in YFPs improved the spectroscopic properties 13

Supplementary Methods **14**
- Plasmid construction and cloning 14
- Plasmid sources and cloning material 14
- Site-directed mutagenesis 14
- Construction and screening of structurally targeted libraries 15
- Error-prone libraries 15
- Protein purification 15
- Spectroscopy 16
- Refolding 16

Crystallography 17
Crystallographic data collection, processing, and refinement 17
Mammalian cell culture and confocal imaging 18
Fluorescence retention, proExM 18
Antibody compatibility 20
Confocal photobleaching 20
Cross-excitation assay 21

Supplementary References 22

Supplementary Figures & Tables 25

Supplementary Table 1. Melting temperature and amino acid sequences of mutants generated in this study, relative to Clover. 26

Supplementary Table 2. Quantification of chemical and thermal denaturation experiments from Fig. 2. 27

Supplementary Table 3. Crystallographic data collection and refinement statistics. 28

Supplementary Figure 1. mhYFP is compatible with commercially available antibodies designed for eGFP. 29

Supplementary Figure 2. Isothermal melting of fluorescent proteins. 30

Supplementary Figure 3. Chromophore environments of hyperfolder proteins. 31

Supplementary Figure 4. Proposed role of C48/C70 and surface hydrogen bonds in avFPs. 32

Supplementary Figure 5. Characterization of cysteine-free mutants and selected library members. 33

Supplementary Figure 6. Mutation map of spectroscopically characterized hyperfolder GFP/YFP mutants. 34

Supplementary Figure 7. Fluorescent protein amino acid sequence comparison to mGreenLantern. 35

Supplementary Figure 8. Statistics tables. 36

Supplementary Video 1. Fluorescence-assisted elution of streptavidin fusion proteins under denaturing conditions. 37

Supplementary Video 2. Fluorescence-assisted elution of streptavidin fusion proteins under denaturing conditions. 38

Supplementary Data

Equilibrium unfolding

We determined the GdnHCl concentration corresponding with the half-maximal fluorescence value ($C_{½}$) for each FP and found that sfGFP showed only a minor rightward curve shift relative to eGFP, with $C_{½}$ = 4.0 and 4.3 M, respectively. Likewise, mClover3 ($C_{½}$ = 3.8 M) was only slightly less stable than eGFP in GdnHCl, whereas mNeonGreen and eYFP showed the lowest values of $C_{½}$ = 2.0 and 1.2 M, respectively. mGL displayed the same curve shape as the control FPs, except that its stability was far greater, at $C_{½}$ = 5.5 M, a dramatic improvement in chemical stability disproportionate to its T_m value (**Fig. 2c**). In the more chaotropic GdnSCN solutions, mGL and hfYFP outperformed other FPs, showing $C_{1/2}$ = 3.2 and 2.3 M, respectively, while $C_{1/2}$ values for eGFP, sfGFP, mClover3, and eYFP were no greater than 0.2 M (**Fig. 2d**) (**Supplementary Table 2**).

Fluorescent proteins with a reduced genetic code

Since the hyperfolder proteins readily tolerate cysteine substitutions that severely diminish eGFP and sfGFP fluorescence, we asked whether the indispensable tryptophan residue that is conserved in all avFPs could now be substituted as well (**Extended Data Fig. 2**). Trp57 stabilizes the avFP hydrophobic core including the central helix “PVPWP” motif [42]. All W57 substitutions produce insoluble, nonfluorescent protein except for the eGFP-W57F mutant that retains only 5% of wild-type eGFP’s brightness[43,44].

We generated W57F point mutants of eGFP, sfGFP, mF4P (**Extended Data Fig. 2**), and hfYFP, and confirmed that the absorbance spectra were unchanged (**Extended Data Fig. 2b**). Like eGFP-C48S/C70V (**Supplementary Fig. 5d**), eGFP-W57F was almost entirely nonfluorescent in E. coli. On the other hand, 30% of sfGFP fluorescence and 84% of hfYFP fluorescence was preserved in their W57F mutants (**Extended Data Fig. 2f**). Here, too, brightness in bacteria corresponded with greater soluble protein production: hfYFP-W57F was 2.5-fold brighter than wild-type sfGFP and 7-fold brighter than sfGFP-W57F (**Extended Data Fig. 2g**). In all cases, the W57F mutation did not change the excitation and emission maxima, QY, or EC (**Extended Data Fig. 2h**).

Even with an 18 amino acid genetic code resulting from three deleterious substitutions (C48S/W57F/C70V), hfYFP-W57F was much more stable than even wild-type sfGFP. hfYFP-W57F persisted in GdnSCN 3.6 M for minutes before denaturing (**Extended Data Fig. 2d**); it remained fluorescent more than 4 times longer than wild-type sfGFP during isothermal melting at 80 °C (**Extended Data Fig. 2c**); and hfYFP-W57F’s melting temperature was still greater than wild-type sfGFP’s (T_m = 89.1 vs. 86.4 °C, respectively) (**Extended Data Fig. 2e**). The data collectively demonstrate that hfYFP is equipped to tolerate major structural perturbations such as those induced by random mutagenesis, due to its assortment of compensatory folding mutations and thermodynamic stability benefits compared to eGFP and sfGFP.

NaOH resistance and development of mhYFP

As mentioned in Results, while experimentally determining FP extinction coefficients (ECs) using the alkali denaturation method[15], we noted that several FPs were not showing the single distinct ~447 nm peak absorbance peak of the alkali denatured chromophore. In contrast to Clover (**Extended Data Fig. 5a**), sfGFP (**Extended Data Fig. 5b**), and all other avFPs we had tested, FOLD6 required 10 min to display the denatured chromophore’s 447 nm absorbance peak without overlap from its 505 nm native peak in this 1 M NaOH solution (pH ≥ 13) (**Extended Data Fig. 5c**). We performed time-course experiments with 8 different FPs and found that Clover, eYFP, and even mGL and hfYFP, denatured right away in 1 M NaOH

(**Extended Data Fig. 5d-g**). By contrast, mF4P (FOLD4-S147P), like FOLD6 (FOLD4-S147P/V68L/L221K) persisted for several minutes (**Extended Data Fig. 5h-i**), suggesting a role of the S147P mutation that we had incorporated into our mutants due to its reported thermostability benefits[21]. mF4Y-SR (FOLD4-S147R/N149Y) also showed delayed denaturation, to a lesser degree (**Extended Data Fig. 5j**), suggesting that mutations to β-strand 7, the most conformationally heterogeneous strand of avFPs[19], were more broadly responsible for the effect (**Supplementary Discussion**).

We attempted to bestow this NaOH resistance into hfYFP using the S147P mutation that benefited the other hyperfolder variants and introduced the V206K mutation to improve monomericity. The V206K mutation improved the OSER score (**Extended Data Fig. 3d-e**) and had a negligible effect on GdnHCl stability when tested in multiple mutants (**Extended Data Fig. 4d**). The S147P mutation conferred mF4P-like NaOH persistence to mhYFP. mhYFP persisted longer than mF4Y-SR in NaOH but not for as long as FOLD6 did (**Extended Data Fig. 5k**). Thus, structure-guided engineering of β-strand 7 yielded monomeric hyperfolder YFP (mhYFP) with enhanced resistance to NaOH and/or extreme alkaline conditions (pH ≥ 13), further demonstrating that peculiar performance features can be engineered into FPs without perturbing spectral properties. The data demonstrate that hfYFP and mhYFP exhibit uncanny stability in diverse chaotropic conditions that rapidly degrade most biological structures in seconds to minutes.

Crystal structures

hfYFP features a chromophore hydrogen bond (H-bond) network indistinguishable from that of eYFP, Citrine, or Venus, besides trivial H-bond distance variation (± 0.1-0.3 Å). In each of those structures, E222 $O_{\varepsilon2}$ is H-bonded to N2 of the chromophore (CRO) imidazolinone ring, while E222 $O_{\varepsilon1}$ is H-bonded to a structurally conserved water molecule, which we will refer to as wat_2. Wat_2 is H-bonded to the backbone amide of L68 and, as we will discuss later, it also connects E222 $O_{\varepsilon1}$ to the Y203 phenolic side chain (**Supplementary Fig. 3a**, yellow). By contrast, this Y203-wat_2-E222 $O_{\varepsilon1}$ connection is broken in mhYFP, with wat_2 situated 3.7 Å away from E222 $O_{\varepsilon1}$, too far to form an H-bond (**Supplementary Fig. 3a**, orange). The electron density of this 1.6 Å resolution structure supports a single E222 conformer whose side chain is rotated so that $O_{\varepsilon1}$ H-bonds to [N2], while $O_{\varepsilon2}$ H-bonds to S205, and S205 H-bonds to wat_1, H148 $N_{\delta1}$, and N146 C=O. Although the chromophore phenolate remains stabilized by H-bonds from wat_1 and H148, this new completed proton wire through E222 (**Supplementary Fig. 3a**) might further stabilize the structure while functionally decoupling the Y203 phenolate from the spectroscopically important E222 side chain. We have not observed this conformation in eYFP (PDB ID: 1YFP)[45], Venus (PDB ID: 1MYW)[46], or Citrine (PDB ID: 1HUY)[47] (**Supplementary Fig. 3e**), perhaps due to the resolution limits of those structures (2.5 Å, 2.2 Å, and 2.2 Å, respectively). Interestingly, we also observe a single E222 conformer in our 1.2 Å FOLD6 structure (**Supplementary Fig. 3b**).

Salt bridges on the hyperfolder protein surfaces

It has been shown that the S30R superfolder mutation enables salt bridges between several residues on the protein surface that contribute to its stability[11], and we observe extensive electrostatic interactions across β strands 1, 2, 5, and 6, in FOLD6, hfYFP, and mhYFP (**Supplementary Fig. 4f-h**). hfYFP shows a single R30 conformation through which four different side-chains are stabilized (**Supplementary Fig. 4g**), whereas mhYFP shows two R30 conformations, shifting the equilibrium more toward stabilization of E32 than D19 (**Supplementary Fig. 4h**). On the other hand, R30 in FOLD6 also shows two conformations, but the rotamers allow R30 to stabilize E32 and D19 via salt bridges and to an H-bond to S28 (**Supplementary Fig. 4f**). These electrostatic surface interactions may contribute to the stability of the hyperfolder FPs.

Supplementary Discussion

Structure-activity relationships in hfYFP, mhYFP, and FOLD6

In our FOLD6 structure (1.21 Å resolution), the H203 side chain is decoupled from a single-conformer E222 by an intervening water molecule, which we refer to as wat_3, bridging the gap between S205 and E222. As we have shown previously in the Clover structure, Clover's E222 $O_{\varepsilon 1}$ associates with the H203 $N_{\delta 1}$ atom through a 2.7 Å H-bond, thereby eliminating the S205-E222 H-bond that sustains the minor protonated chromophore population observed in eGFP high-resolution structures (PDB: 4EUL)[42], which manifests as a 405 nm absorbance band[23]. In FOLD6, affinity of E222 for neighboring wat_3 (3.1 Å distance) has withdrawn E222 from H203 and brought it closer to β-strand 11, eliminating this Clover-like H-bond (**Supplementary Fig. 3b**).

Consequently, H203 is shifted even farther away from E222 relative to Clover, placing the H203 $N_{\delta 1}$ atom directly above and tilted 21° into the Y66 centroid relative to the ring edge nearest F165, a parallel-displaced π-stacking interaction almost identical to that found in the disulfide-oxidized structure of roClover0.1 (Clover-S147C/Q204C is a redox biosensor template we reported previously[23]). Perhaps for this reason, the FOLD6 chromophore is far more planar than hfYFP's where, by contrast, the centrosymmetric π-stacking of Y66-Y203 leaves the Y66 ring noticeably rotated about the methylene bridge relative to the imidazolinone moiety to achieve greater coplanarity with the Y203 ring. Deviations from planarity between the chromophore imidazolinone and phenolate moieties are well known to decrease quantum yield[30,48].

The highly off-center H203 orientation relative to Y66 considerably widens a separation between β-strands 7 and 8 in FOLD6 through steric effects on H203. Compared to Clover, the H203 side chain has shifted 0.5 Å away from E222 and toward V150, pushing the V150 side chain and local β-strand 7 backbone 1.0-1.3 Å outward and away from H203. This new V150 position forces the F165 side chain down 0.6 Å toward H181 and the F165 C_α carbon 1.0 Å outward, while the neighboring N164 carbonyl rotates more than 25° away from the Y151 amide, severing the β-strand H-bond between the two (linear distances of 3.1 Å in sfGFP and 5.1 Å in FOLD6). hfYFP shows similar conformational changes that fall between those of sfGFP and FOLD6. We speculate that these changes might facilitate chromophore maturation by increasing the porosity of barrel sections adjacent to catalytically important residues during protein folding and chromophore cyclization steps.

In all three of our structures, the C48 and C70 side chains are replaced with serine and valine, respectively, and the same conformations are observed in all. The C48S substitution eliminates sulfur-aromatic and vdW interactions with W57 and F27 and weakens or extinguishes numerous long-range weakly polar interactions with the loop main-chain atoms, instead producing a 2.3 Å H-bond between the S48 side-chain and G51 carbonyl (**Supplementary Fig. 4b**). On the opposite side of the chromophore and along the central helix, C70 coordinates vdW forces and sulfur-aromatic interactions with F8, F71, and Y92. These interactions are eliminated in the C70V mutants and are largely replaced by vdW forces, with the shortest distances observed between V70 and F8 side-chains (**Supplementary Fig. 4c**). Overall, the C48S and C70V mutations may destabilize these micro-regions, although computational comparisons would help verify this assertion.

Hyperfolder YFP combines two distinct features of Venus (PDB: 1MYW)[46] and Citrine (PDB: 1HUY)[47]: the Q69M mutation, and the F46L/F64L mutations, respectively. In hfYFP and Venus, the F46L/F64L mutations eliminate the antiparallel π-π stacking interaction present in eGFP and the closely related structures, eYFP and Venus. Consequently, the smaller alkyl side-chains of L44, L42, and L220, among

other residues that line the protein's hydrophobic core (approximately bordered by N121), shift to occupy the space (**Supplementary Fig. 3c**).

The Q69M mutation from Citrine substitutes a polar side chain with the hydrophobic yet weakly polar and larger methionine, which packs more efficiently into the chromophore cavity and is known to improve chromophore maturation[47]. The conformations of M69 in hfYFP and Citrine structures overlap due to extensive side-chain packing (**Supplementary Fig. 3c**): M69 is situated directly between three aromatic residues, not including the chromophore's π-conjugated system itself, and M69 shows notable orientational preference for the δ^+ ring edge of F84, whose centroid is located approximately 5 Å away with the δ^- M69 sulfur atom and a π-cloud presumably oriented 90° relative to the ring (**Supplementary Fig. 4a**). This configuration indicates a sulfur-aromatic interaction that should contribute stabilizing energy greater than the sum of the vdW forces[49]. Additionally, the Y203 centroid and the chromophore imidazolinone ring are approximately 6 Å from M69 S_δ, suggesting that the sulfur atom's lone pair electrons may stabilize those features as well. Altogether, M69 serves a variety of functions that the wild-type glutamine side-chain cannot, including greater hydrophobic packing and long-range stabilizing interactions with the aromatic residues that may improve the FP's overall stability.

Additional observations related to chromophore maturation

Our spectroscopically characterized library data allow us to generate new conclusions about existing structures, and we mention several examples here. One is that mClover3's slow maturation compared to Clover's ($t_{½}$ = 37 min vs. 15 min) is primarily due to its G160C mutation, and perhaps partly due to V206K reversion (compare the maturation rate of $mF2BK^{DMD}$ to mGL's in **Extended Data Table 1**; the difference is the V206K mutation). It does not appear due to N149Y, which improved maturation in at least two of our GFPs. Additionally, we can conclude from our data and from a previous study[50] that the V68L mutation that differentiates mF4P from FOLD6 (besides the phenotypically neutral L221K monomerizing mutation[51]) is completely responsible for delaying FOLD6 maturation 2-fold relative to mF4P (**Extended Data Table 1**).

We speculate that the F46L mutation of Venus, which is reported to improve the maturation rate of YFPs[52], may serve to counteract the maturation-delaying effect of V68L, perhaps alongside the F64L mutation that serves that role[46]. This may occur through positional adjustment of the L68 main-chain and its proximity to wat_2 (**Supplementary Fig. 3a**) and through a chain reaction of alkyl side-chain adjustments in the hydrophobic region of the protein, part of which is depicted in **Supplementary Fig. 3c**, clockwise from E222 to N121 (the L68 main-chain is pictured, but the side-chain was omitted for clarity in the context in which the figure is discussed).

Mechanisms for maturation enhancement by the I167T mutation

Our mF4P and FOLD6 point mutants allow us to define structure-function relationships resulting from the I167T mutation and, more generally, the data suggest a potential tradeoff between stability and solvent accessibility that accelerates the chromophore cyclization rate.

The I167T mutation has been known to diminish the 405 nm absorbance (A-band) that arises from the protonated chromophore species[53]. Most of our mutants carry the I167T mutation (**Supplementary Table 1**) based on our hypothesis that it contributes to mGL's rapid chromophore maturation, an assertion that is supported by previous observations[54,55]. Indeed, most of our GFP mutants mature very rapidly. mF4Y-SR exhibits the fastest maturation time that we've recorded, requiring only 6 min to reach half-maximal fluorescence intensity after ambient air introduction (**Extended Data Table 1**).

Structurally, the I167T mutation in FOLD6 enables a 2.9 Å H-bond between T167 $O_{\gamma1}$ and H169 $N_{\varepsilon2}$, shifting the imidazole side chain 1.0 Å upward toward T167 and increasing the channel diameter by 1 Å. A new H_2O molecule is found H-bonded to the T167 side chain in the polar core of the protein adjacent to H181, which is now tilted 30° directly toward T167, but only in the FOLD6 structure (the others contain Ile167). Distances between the nearest H_2O molecules (≤ 2.0 Å) in our 1.2 Å resolution structure indicate more heterogeneous occupancy than the rigidly constrained water molecules lying closer to the central α-helix. Altogether, this new configuration eliminates the hydrophobic barrier of isoleucine's side-chain and enables an uninterrupted H-bond circuit through H169 all the way into the protein's polar core (**Supplementary Fig. 3d**).

Although surface contours do not explicitly map an open channel through T167-H169 in the mature FOLD6 protein (or in any avFP, to our knowledge), it is probable that the channel is sufficiently polar to conduct water molecules at least during the final steps of folding and barrel closure[56] while chromophore cyclization proceeds as a parallel process[57]. Such hydrated channels are of demonstrable importance to the chromophore maturation of TurboGFP, an FP derived from *Pontellina plumata*[58]. Water exchange may also occur in the mature protein, as suggested by molecular dynamics (MD) simulations of the avFP β-barrel, and may influence its function[59]. MD simulations of our crystal structures that probe organic solvent exchange at the β-barrel interface would be valuable for understanding the susceptibility or resilience of FPs to various tissue clearing reagents or fixatives, such as those we tested in **Fig. 3**, and might lead to further optimized variants.

We probed structure-function implications of the I167T mutation experimentally. Introducing H169L to disrupt the described T167-H169 H-bond (**Supplementary Fig. 3d**) increased the pK_a from 5.9 to 6.7 and decreased cellular brightness by 50%, a substantial drop that can be primarily attributed to the pK_a shift. From a structural standpoint, the aliphatic leucine side-chain that replaces histidine increases the hydrophobic character of the hydrated tunnel and opposes the hydrophilic advantage (including the H-bond) conferred by the Thr167 side-chain. Our experimental data support this assertion, as reverting T167 to wild-type I167 in mF4P (producing mF4Pti) doubled the maturation latency and halved the cellular brightness (**Extended Data Table 1**). Although the QY improvement from 0.75 to 0.77 in mF4Pti relative to mF4P is within the experimental error range, it may be significant, and it is conceivable that Ile167 could stabilize the chromophore and F165 through vdW forces that rigidify this region and decrease nonradiative decay of the excited state. If so, the stabilizing benefit of Ile167 may come at the cost of a maturation advantage afforded by smaller residues such as Thr.

If vdW contacts between the chromophore and F145 are useful for decreasing nonradiative quenching of the excited state, which is likely, then that might also partly explain the decreased QY of hfYFP relative to the GFPs, since the F165 side chain is considerably shifted away from the chromophore in hfYFP and mhYFP, and even more so in FOLD6, yet the chromophore remains in the same position. Decreased friction from longer and weaker vdW associations with F165 might increase the probability of nonradiative de-excitation through chromophore twisting motion in hfYFP. Chromophore planarity might also play a role.

As far back as 1994, Roger Tsien's group suggested that the I167T mutation stabilizes the B-state, which at the time was suspected but not definitively demonstrated to represent the deprotonated chromophore[53]. Later work has suggested that the tip of Ile167 $C_{\delta1}$ makes an unfavorable contact with the chromophore phenolate that stabilizes the A-state and that this interaction is relieved by Thr167, thereby enhancing the B-state. The authors observed the same T167-H169 H-bond that we report here in several I167T-containing structures[60]. In addition to these observations, we propose based on our crystallographic and functional data

that I167T shifts the chromophore equilibrium to the predominantly deprotonated state by stabilizing H169 through the T167-H169 H-bond and opening the polar channel to exert long-range effects into the protein's hydrophilic core (**Supplementary Fig. 3c-d**), which also accelerates chromophore maturation.

Presumably, E222 remains protonated (and the chromophore deprotonated) because the FOLD6 H-bond network minimizes environmental changes to the proton wire that would alter the protonation status of either residue, with interactions resulting from the I167T mutation further conferring a low pK_a (**Supplementary Fig. 3d**). We interpret this function of I167T through the pK_a-reducing effect that decreases the A-band—shifting the pH curve—while the hydrated channel from outside the protein into the chromophore environment accelerates chromophore maturation during folding.

This explanation may account for the marginal EC drop in FOLD6-H169L relative to FOLD6 (118 to 108 $mM^{-1} cm^{-1}$), as well as the lack of benefit to our YFPs, all of which contain the Q69M mutation (relative to eGFP, eYFP, and Venus) that strengthens the chromophore's hydrophobic packing as discussed and leaves little room above the chromophore for water molecules to reside (**Supplementary Fig. 4a**), in contrast to the open cavity in FOLD6 (and in the Clover structure[23]) that originates from Clover's Q69A mutation and influences the proton wire of both (**Supplementary Fig. 3b**). This key structural difference between the GFPs with A69 and YFPs with M69 may explain why reverting Thr167 in mfoxY to Ile167 in mfoxYti (hyperfolder YFP)—like the T167I reversion that produced mF4Pti—increased the QY and EC greatly without appreciably changing the pK_a (**Extended Data Table 1**): the hfYFP chromophore environment is already tightly packed and more hydrophobic than those of H203-A69 GFPs such as Clover, mF4P, and mGL.

Hydrophobic packing in the hfYFP chromophore environment

Late during the course of our study, we became aware of another protein that has been engineered for tissue clearing applications: muGFP, or sfGFP-Q69L/N164Y/F223D[61]. Using their muGFP crystal structure (PDB: 5JZL), the authors concluded that elimination of a water molecule in the chromophore environment, and greater hydrophobic packing, among other reasons, contributed to the improved reported stability of muGFP relative to eGFP.

Likewise, we have concluded in our study that the Q69M mutation in hfYFP (relative to eGFP), would improve hydrophobic packing within the protein not just by eliminating water molecules, but via the potentially stabilizing effects of sulfur-aromatic interactions of Met69 with Phe84, Tyr203, and the chromophore itself (**Supplementary Fig. 4a**). Methionine-aromatic interactions have been described and quantified, and in many cases these interactions play an important and unique role in protein structure stabilization[62,63].

Further efforts to develop FPs for tissue clearing and super-resolution applications may benefit from structural examination of the chromophore packing environment and identification of suitable templates, such as hfYFP and LSSmGFP, that can tolerate extensive hydrophobic interactions while maintaining the specific H-bond networks that are critical for producing the desired fluorescence spectra (**Supplementary Fig. 3a-b**).

Discovery of the S147R mutation

While mutating mF4Y (**Supplementary Fig. 6**) to produce an S147P variant, we fortuitously identified a *de novo* S147R mutation that dramatically accelerates chromophore maturation. mF4Y-S147R has the fastest maturation rate of any of our mutants except mF1Y (contains N149Y) and $mF2BK^{DMD}$ (contains N149K) with half-times of 6 min and 7 min, respectively (**Extended Data Table 1**). During our study, the

S147R mutation was independently reported by a research group screening for photostable avFP variants, although an effect on maturation was not described[64]. Structurally, the S147R mutation replaces the small polar serine side chain with a volumetrically larger but also more flexible charged residue in a section of the barrel that is well-described as the most heterogeneous[19]. It is conceivable that the S147R side chain could form cation-π stacking interactions with Y149 in the mF4Y protein or various electrostatic interactions with neighboring residues to offer new conformational options during protein folding. In any case, our data demonstrate that the composition of β-strand 7 is an integral feature of the chromophore maturation process.

A third water molecule in the chromophore proton wire

The FOLD6 structure reveals a rare water molecule, wat_3, that is also found in roClover0.1 (structure solved to 1.3 Å resolution), but only in the B-chain. It doesn't appear in Clover, perhaps due to limitations of the structure's solved resolution of 2.4 Å. The presence of wat_3 in these examples is interesting because, to our knowledge, the only other structure that renders wat_3 is the 1.7 Å resolution off-state of Dreiklang (Citrine-V61L/F64I/Y145H/N146D) where it is referred to as "wat_c" by the authors. It does not appear in the on-state (2.0 Å resolution) or the equilibrium state structures[65]. Whether or not wat_3 plays a role in the chromophore H-bond network in Dreiklang is not entirely clear, but in the FOLD6 structure—which shows a single E222 conformation—the proximity of wat_3 to the polar side-chains of S205 and E222 appears to form two H-bonds to stabilize the proton wire and, presumably, maintain a protonated E222 side-chain and deprotonated chromophore (**Supplementary Fig. 3b**).

Slow denaturation in NaOH

We are aware of one example in the literature of comparably slow denaturation in the 1 M NaOH solution (**Extended Data Fig. 5**) that is used to obtain extinction coefficient (EC) values (**Supplementary Methods**): the eqFP611 template[66] from which mRuby was engineered[67]. Of course, there are considerable differences between anemone- and jelly-derived FPs, but it appears there is a functional similarity that suggests a common structural element. Our data suggest that rigidification of β-strand 7, perhaps through the S147P mutation (mF4P, FOLD6, and mhYFP all feature it), contributes to the extended NaOH tolerance. Upon aligning the hfYFP and mRuby structure (PDB: 3U0M)[68], there is a proline residue at the equivalent mRuby position, within a tighter β-strand 7 turn that may be expected to decrease the strand's conformational heterogeneity. Further studies of the hfYFP structure, including molecular dynamics simulations, could lend deeper insight into these peculiarities and further advance the engineering of new FPs for biotechnological applications.

Supplementary Note

Library development

We first created cysteine-free mutants of a variety of *Aequorea victoria* fluorescent proteins (avFPs) and compared their brightness, thermostability, and spectroscopic characteristics. Clover[16] tolerated cysteine substitution better than eGFP and sfGFP (**Supplementary Fig. 5d**). Whereas the C48S/C70V mutations eliminated eGFP's fluorescence in cells, Clover-C48S/C70V was brighter than wild-type Clover, and sfGFP-C48S/C70S (moxGFP)[13] was almost 50% dimmer than sfGFP (**Supplementary Fig. 5b**). The spectroscopic properties of each cysteine-substituted mutant were essentially unchanged (**Supplementary Fig. 5d**), suggesting that the brightness in cells results from better expression, folding, maturation, and/or stability.

We expanded the library built on Clover-based mGreenLantern (mGL)[6] by introducing C48S/C70V substitutions, followed by combinations of folding mutations that we rationally selected based on extensive analysis of structure-activity relationships (SARs) in avFPs. The unusually long persistence of mGL in guanidinium hydrochloride (GdnHCl) was one of the defining stability metrics that distinguished it from sfGFP and underlie its improved performance. In this vein, we screened FPs in kinetic unfolding assays and determined their melting temperatures (T_m) to identify mutants with improved thermodynamic stability. We compared individual and concerted folding mutations in this background in a generally stepwise manner (**Supplementary Table 1** and **Supplementary Fig. 6**), relying on spectroscopic characterization (**Extended Data Table 1**), thermostability measurements (**Supplementary Fig. 5e-f**), FP brightness in live human cells ("cellular brightness") (**Supplementary Fig. 5g**), and GdnHCl stability (**Extended Data Fig. 4c**) to identify trends that would guide the ensuing library design steps. Individual and concerted sets of mutations conferring unique properties could often be transferred into other templates.

The most stable mGL variant generated from this effort, hyperfolder YFP (hfYFP), has a melting temperature of 94.2 °C, an improvement of 21 °C above eYFP's T_m and 8 °C above sfGFP's. The majority of mGL variants showed superior stability in 6.3 M GdnHCl solutions relative to sfGFP, persisting for over 1 hr before falling to half-initial fluorescence values. Intriguingly, rather than falling, hfYFP's fluorescence instead increased 50% upon exposure to GdnHCl 6.3 M and remained constant for over 10 hours, the full duration of the experiment (**Extended Data Fig. 4c**). Cysteine-containing mutants such as sfGFP failed to refold after denaturation (**Extended Data Fig. 4a**) unless reducing agent was present in solution (**Extended Data Fig. 4b**), whereas the cysteineless variants fully refolded under both conditions.

Development of cysteine-free fluorescent proteins

The two highly conserved cysteine residues in avFPs are spatially separated and cannot form a disulfide bond in the native fold (**Supplementary Fig. 5a**), but they enable an important misfolding pathway through interchain disulfide bond formation while the nascent polypeptide is processed through the secretory pathway[13]. Counter-intuitively, nearly all substitutions to C48 and C70 result in nonfluorescent protein[69], suggesting that cysteine substitution may destabilize the protein structure, and/or the folding process through a disulfide-independent mechanism. The best-tolerated substitutions appear to be C48S[13,69,70] and C70V[25,47]. Although introducing the "cycle-3"[71] or "superfolder" mutations[11] can partially rescue brightness[13,25,47], double mutants rarely retain their original fluorescence intensity for reasons that are not known.

We produced C48S/C70V double mutants of eGFP, Clover[16], and mF1Y (an intermediate from mGreenLantern development) (**Supplementary Fig. 5b-d**), and compared them to the original proteins.

We also included the human codon-optimized sfGFP-C48S/C70S variant, “moxGFP”[13], to accompany sfGFP. Whereas *E. coli* colonies expressing eGFP-C48S/C70V were practically nonfluorescent, the Clover-C48S/C70V colonies appeared minimally affected. To quantify the brightness of C48S/C70V double mutants in cells, we used an established co-expression strategy that generates each FP and an mCherry in a roughly equimolar ratio, allowing normalization to the red fluorescence and thereby controlling for transfection-related variability[41]. Consistent with our observations in *E. coli*, eGFP-C48S/C70V was nonfluorescent in human cells, while moxGFP (sfGFP-C48S/C70S) was slightly dimmer than eGFP and half as bright as sfGFP. Interestingly, Clover-C48S/C70V was brighter than Clover in all three human cell lines tested, displaying 2.7-fold greater brightness than eGFP, compared to 2.2-fold for Clover relative to eGFP. mF1Y and its double mutant were both 3.5-fold brighter than eGFP (**Supplementary Fig. 5b**).

To assess the impact of cysteine substitutions on protein stability, we experimentally determined FP melting temperatures (T_m) and found that the C48S/C70S mutations of moxGFP eliminated the thermodynamic stability benefit conferred by the superfolder mutations: moxGFP’s T_m of 79.5 °C is approximately the same value as eGFP’s and Clover’s. Interestingly, Clover-C48S/C70V displayed a minor secondary melt peak at 89.6 °C, higher than sfGFP’s single peak of 86.4 °C. Clover-C48S/C70V’s primary T_m peak decreased by only 3 °C compared to the 7 °C drop seen between sfGFP and moxGFP. The secondary peak was visible as a distinctive phase that was absent in the other FPs (**Supplementary Fig. 5c**). The data suggest that a unique structural change occurs at high temperature in Clover-C48S/C70V that stabilizes the protein and/or shields the chromophore from quenching.

There were no obvious differences in typical spectroscopic characteristics that could satisfactorily explain the cellular brightness and T_m differences between the cysteine-replaced variants and their parents (**Supplementary Fig. 5d**), indicating that folding and/or chromophore maturation processes might be hindered. Indeed, we found that chromophore maturation occurs 35% more slowly in moxGFP than in sfGFP, while Clover-C48S/C70V matures at half the rate of Clover (**Supplementary Fig. 5d**). The data imply that delayed chromophore maturation in these mutants arises from thermodynamically unfavorable interactions during the protein folding process that ultimately yield a destabilized final structure.

Although it might appear that eliminating undesirable interchain disulfide bond formation by removing cysteines solved one folding problem and introduced another, the presence of a second melt peak in Clover-C48S/C70V raised the possibility that the avFP thermodynamic equilibrium could be shifted toward a more stable final conformation using structure-guided engineering, perhaps without even modifying intrinsic spectral properties.

To test that hypothesis, we applied the C48S/C70V mutations to various mGL mutants and re-introduced mutations that we had removed previously while optimizing for brightness, including those most closely attributed to the stability of “superfast GFP” variants: mutations K101E, N105Y, E124V, G232D, and D234N[40], as well as the “superfolder” A206V mutation. Some mutations, such as F223R, we reverted back to wild-type (**Supplementary Fig. 6**), since the L221K mutation is enough to monomerize eGFP[51] and we did not know what role F223 might play in protein stability.

Introducing C48S/C70V into F3C and related variants produced a series of FPs with T_m values ranging from 84-88 °C, like sfGFP’s (T_m = 86.4 °C) (**Supplementary Table 1**), despite the absence of cysteine residues, which we have shown are detrimental to the thermodynamic stability of eGFP-C48/C70V and moxGFP (**Supplementary Fig. 5c**). The foundational protein of our “hyperfolder” library was FOLD4, a C48S/C70V mutant that we constructed as a hybrid template containing the superfolder GFP[11] and superfast

GFP "P7"[40] mutations in the Clover background (characterized by T65G/Q69A/T203H mutations relative to sfGFP), along with several mutations from Emerald that improve *E. coli* colony brightness[55] (**Supplementary Table 1** and **Supplementary Fig. 6**). Introducing the V68L mutation common to YFPs raised the T_m of a mutant coded "FOLD6" to 90.0 °C, approximately the value of the second melt peak in Clover-C48S/C70V.

Role of V68L in Tm and maturation rate

Our finding that V68L raised thermostability above the apparent local maximum of T_m = 84-88 °C for the explored sequence space of the FOLD4 variants marked a turning point in our effort to develop stability-enhanced FPs. The V68L mutation is widespread in avFPs because it improves the chromophore oxidation rate of YFPs (GFP-T203Y variants) through structural rearrangements including H-bonding of the central α-helix main chain to structural water molecules involved in the proton wire[46]. Moreover, V68L was present in the superfast GFP library template[40] that influenced our development of this series of proteins, perhaps indicating a beneficial role in folding and/or structural stability.

Consistent with a previous report suggesting a photostability improvement at the cost of maturation rate[50], V68L did indeed slow chromophore maturation when introduced into F4P to produce FOLD6, doubling the latency to completed chromophore cyclization compared to mF4P, to 25 min. That rate, however, was still faster than the maturation rate of eGFP, sfGFP, moxGFP, mClover3, and eYFP, among others **(Extended Data Table 1**). The V68L mutation of FOLD6 also improved the rate of refolding to the half-maximal value relative to mGL, but with reduced slow-phase kinetics of its double-exponential curve. Performing the refolding experiment without reducing agent DTT completely prevented all the cysteine-containing FPs (eGFP, sfGFP, Clover, and mGL) from refolding. The refolding rates and curve fits were practically identical in the DTT(-) condition (**Extended Data Fig. 4a-b**), confirming that cysteine oxidation was responsible for the refolding deficit observed under these *in vitro* experimental conditions.

Inspiration for the YFP templates

Suspecting we had reached a T_m ceiling in the Clover-type GFP background (with His203), we generated YFPs carrying Tyr203. We blended features of Citrine[47], Venus[46,52], and YPet[72] in a structure-guided manner, excluding any mutations that we deemed superfluous or application-specific, such as the various mutations in YPet that enhance FRET through dimer interface modifications (e.g., S208F and V224L were excluded). Introducing the F46L/A69M/H203Y/D234N mutations into FOLD6 produced the first of several YFPs with melting temperatures exceeding 90 °C (**Supplementary Fig. 5e-f**) and cellular brightness values equal to or greater than eYFP's (**Supplementary Fig. 5g**).

In addition to the FOLD6 (F4P-V68L) experimental data (**Extended Data Table 1**), several extant FPs inspired the construction of our first YFP template, foxY. Citrine is a YFP with low acid sensitivity (pK_a = 5.7), improved refolding, and greatly reduced chloride sensitivity compared to eYFP[47] and to Venus[73]. Intriguingly, Citrine was reported to tolerate C48L/C70V substitutions, although the mutant was not extensively explored[47]. Additionally, we recognized that T-Sapphire, a well-folded GFP with a large Stokes shift (LSS), features the Q69M and C70V mutations, the latter of which reportedly originated from a spontaneous PCR error[25]. The presence of Q69M/C70V in the well-folded T-Sapphire and the possibility that these mutations would be compatible with Citrine instilled further confidence that our proposed YFP template would at least tolerate cysteine substitution.

We included the F46L mutation from Venus that purportedly improves chromophore maturation rate and refolding kinetics[52] of YFPs but not GFPs[25,50,52], perhaps due to the identity of the position 64 side chain

and/or the concerted effects of multiple "YFP-type" background mutations including T65G/V68L/T203Y, which we included as well. T203Y is the defining mutation that produces the bathochromic shift separating the GFP and YFP spectral classes[45].

Lastly, we re-introduced a mutation that we had tested previously in our early GFPs, D234N, which was reported in a FRET-optimized bright YFP, YPet[72]. The D234N mutation was later identified independently alongside G232D in one of the "superfast GFP" mutants[40], reinforcing our impression that the avFP C-terminus, which is usually unstructured in X-ray diffraction data, might play some role in protein stabilization. The mutations F46L/V68L/A69M/H203Y converted FOLD6 into foxY, which we then monomerized using L221K and F223R (to produce mfoxY), supposing that including both mutations would be advantageous in the more dimer-prone A206V background[15]. As expected, the foxY proteins showed YFP-type excitation and emission maxima, but the quantum yield (QY) was 0.51, below the value of most avYFPs, including eYFP ($\phi = 0.60$) (**Extended Data Table 1**). Therefore, we sought to improve the QY.

Thr167 reversion to Ile167 in YFPs improved the spectroscopic properties

By sequence and structure alignment, we could find no avYFP containing Thr167, and moreover, we suspected that Ile167 would promote favorable vdW interactions with the chromophore and with nearby F165 in the packed protein core. Re-introducing the wild-type Ile167 into mfoxY to produce mfoxYti (hfYFP) increased the QY by 20% to 0.61 and the EC to nearly 120,000 M^{-1} cm^{-1} without changing the pK_a of 5.5 (**Extended Data Table 1**) or the GdnHCl resistance (**Extended Data Fig. 4c**). This reversion to Ile167 accelerated chromophore maturation by another 16%, to 21 min, which is faster than eGFP and eYFP's ($t_{½}$ = 28 and >37 min, respectively). It also increased the T_m from 92.8 to 94.2 °C (**Extended Data Table 1**). All YFPs from this series displayed cellular brightness values 2- to 3-fold greater than eGFP's. We named the mfoxY-T167I variant 'hyperfolder YFP' (hfYFP) and spectroscopically and functionally characterized it (**Fig. 1**).

hfYFP is mGL-F46L/C48S/V68L/A69M/C70V/K101E/T105Y/K149N/T167I/H203Y/K206V/D234N.

Amino acid alignment can be found in **Supplementary Fig. 7**.

Supplementary Methods

Plasmid construction and cloning

All FPs in this study (besides mScarlet-I) were designed to maintain the seven avFP-type N-terminal amino acids, MVSKGEE, encoded by nucleotide sequence ATGGTGAGCAAGGGCGAGGAG. Likewise, the C-terminal avFP amino acids, GMDELYK (nucleotides GGCATGGACGAGCTGTACAAG), were maintained in all FPs except those carrying the G232D and G232D/D234N mutations (resulting in GACATGGACGAGCTGTACAAG and GACATGAACGAGCTGTACAAG, respectively). See **Supplementary Fig. 7** for amino acid alignment. PCR primers 21 nucleotides in length were designed using those sequences in the appropriate orientation to amplify the FP gene or, alternatively, the entire host plasmid as a linearized empty vector. This primer design strategy maintains proper stop codon placement. FP gene amplicons and linearized empty vectors with compatible termini were amplified, purified, and quality-controlled so that any combination of FP gene and vector could be cloned on-demand from the same insert/vector stocks using restriction site-independent isothermal "Gibson" assembly of the overlapping DNA fragments[74]. This approach works well for simple plasmids like pBAD and pcDNA3.1 but should not be used for plasmids with repetitive sequences or complex secondary structure, such as viral vectors. Consequently, cloning into the adeno-associated virus (AAV) expression vector pAAV-CAG-FLEX was performed using standard T4 ligation between the *BamHI*/*EcoRI* sites.

Fusion constructs depicted in Figure 5g were cloned into a pET28a vector modified to remove the N-terminal thrombin cleavage site while preserving the N-terminal hexahistidine (His_6) tag. The full pET28a-hfYFP plasmid was amplified to linearize it between the 3' end of hfYFP and the vector backbone. The oligonucleotides provided long overhangs coding for the first half of a linker at the 3' end of the hfYFP sequence, while the fusion protein genes (mScarlet-I, *Bacillus circulans* xylanase, or streptavidin) were amplified using oligos to complete the linker (GSAGSAAGSGEFENLYFQGH) at the 5' end of the gene and hybridize with the pET28a backbone on the 3' end. The complete circular plasmid was generated from these two fragments using Gibson Assembly.

Plasmid sources and cloning material

All plasmids used in this study were generated from plasmids or gene synthesis products described in Campbell et al. (2020)[6], except for those listed in this section. pEGFP-N1-moxGFP was obtained from Addgene (#68070)[13]. pCytERM_mScarlet-i_N1 was obtained from Addgene (#85066)[30]. The streptavidin core domain sequence, constituting amino acids 13-140 of the native protein and responsible for its activity[31], was synthesized without further codon optimization. *Bacillus circulans* xylanase (synonymous with *Niallia circulans* endo-1,4-beta-xylanase) was synthesized from UniProtKB/Swiss-Prot: P09850.1 amino acid sequence using a bacterial codon set (Eurofins Genomics).

Site-directed mutagenesis

Site-directed mutagenesis was performed using the QuikChange (Stratagene) method with *Pfu* polymerase (Agilent), or using the QuikChange Lightning Multi Site-Directed Mutagenesis Kit (Agilent) as described[6]. Non-phosphorylated mutagenic primers were designed to introduce the most abundant human codon for the target amino acid. Degenerate codon selection for multi-site mutagenesis were performed manually or facilitated by the SwiftLib program[75]. Full gene codon optimization was not performed and the nucleotide background ultimately originates from Clover[16], which is based on eGFP. For the Clover nucleotide sequence, refer to Addgene #215680.

Construction and screening of structurally targeted libraries

To generate small structurally targeted libraries to produce 405 nm excitable FPs, specific residues were selected for mutagenesis based on the hfYFP crystal structure as described in **Fig. 5a-b** and **Supplementary Figs. 3-4**. When modifying multiple adjacent codons (e.g., to mutate β-strands 10 and 11), primers were designed to amplify large sections of the FP gene with overhangs at junctions between the segments containing the standard or degenerate codons targeting those sites, followed at the 3’ end by a homology arm for the adjacent gene fragments amplified in separate reactions. The gene fragments containing these degenerate codons at the junctions were stitched together by overlap-extension PCR[76], gel purified, and cloned by isothermal assembly[74] into a PCR-amplified linear pBAD vector for transformation and expression in *E. coli*.

Mutated FP genes were transformed into TOP10 competent cells and grown at 37 °C on LB agar plates supplemented with carbenicillin (100 µg/µL) and 0.02% arabinose. The next day, colonies were screened by eye using alternating 405 nm and 470 nm LED illumination while fluorescence was observed through amber long-pass filter goggles (Invitrogen #S37103 or ThorLabs #LG10). Colonies that glowed brightest under 405 nm excitation while showing minimal fluorescence under 470 nm illumination were picked into sterile 96-well deep-well blocks containing 1 mL LB medium supplemented with ampicillin (100 µg/µL) and 0.2% arabinose. The culture blocks were sealed with a breathable adhesive (EasyApp Microporous Film, USA Scientific #2977-6202) to permit air and gas exchange while minimizing evaporation, and cultures were grown at 37 °C with 275 rpm shaking for 16-18 hr. The following day, 100 µL overnight culture was pipetted into black clear-bottom 96-well optical plates (Corning) for first-pass excitation scans, and the ratio of 405 nm to 488 nm excitation was scored. Soluble protein from cultures with the greatest 405/488 nm ratio scores were extracted using B-PER II reagent (Thermo Scientific), re-scanned for confirmation, and the same lysate was then used for kinetic unfolding screens.

Error-prone libraries

Error-prone libraries were generated using the staggered extension process (StEP)[27] with some modifications. Plasmid DNA from 12 mutants from the LSSA12 library was pooled and diluted 50% with hfYFP-V206K/G65S/Y203I plasmid. To increase error rate, 0.5 $MnCl_2$ weas added to this *Taq* polymerase-based PCR reaction containing 33 ng total template DNA and 30 pmol of forward and reverse primer (flanking the complete FP gene). The thermal cycling program consisted of denaturation at 94 °C for 30 s and combined annealing/extension steps at 55 °C for 5 s, repeated 100 times before the reaction was cooled to 4-10 °C. The PCR product was digested for 5 min using *DpnI* to eliminate residual parental plasmid, and the DNA was isolated using spin columns (Thermo Scientific #K0702) to remove reaction components. A single 720 bp band was observed upon analysis by gel electrophoresis. To generate additional product for cloning and storage, the StEP library was gel extracted and amplified using Phusion HS II polymerase (Thermo Fisher) with the same flanking primers. The reaction product was cloned without further purification by isothermal assembly into PCR-amplified linear pBAD vector and expressed and screened as described.

Protein purification

Fluorescent proteins were purified as described[6] using Ni-NTA chromatography, without lysozyme or DNAse for the large scale 500 mL preparations. Samples were dialyzed into TN buffer (50 mM Tris-HCl, 150 mM NaCl, pH 7.5), concentrated when necessary using 3K or 10K MWCO centrifugal filter units (MilliporeSigma Amicon), flash frozen in TNG buffer (TN plus 10% glycerol) using liquid N_2, and stored at -80 °C. Protein for X-ray crystallography was prepared as described[23] using pET28a-hfYFP, pET28a-

mhYFP, and pET28a-FOLD6 vectors and purified by Ni-NTA chromatography. After overnight dialysis in TN buffer, gel filtration chromatography was performed using a HiPrep 16/60 Sephacryl S-200 HR column at a flow rate of 0.5 mL/min and fractions were collected while monitoring absorbance at 280 nm. The eluted FPs were filtered through an 0.22 µm membrane and concentrated to 20-40 mg/mL using Amicon centrifugal filter units (EMD-Millipore).

Spectroscopy

Unless otherwise stated, all optical assays were performed using a BioTek Synergy H1 microplate reader. Samples were dispensed into black clear-bottom 96-well assay plates (Corning Costar) or into 1 cm path length micro-volume quartz cuvettes (Hellma Suprasil) placed horizontally in a BioTek Take3 microplate. Stability assays took place in TNG buffer (50 mM Tris-HCl, 150 mM NaCl, 10% glycerol, pH 7.5) and used detection settings $\lambda_{ex}/\lambda_{em}$ = 495/525 nm (or 405/525 nm for the LSS FPs), unless otherwise specified.

Chromophore pK_a measurements were determined as described[15]. Chromophore maturation rate was determined as described[6]. Full absorbance spectra were collected in quartz cuvettes from 250-650 nm in 1 nm steps.

Extinction coefficients (ECs) for were determined using alkali denaturation[15]. Protein sample was diluted 1:1 into 2 M NaOH in a quartz cuvette, mixed rapidly, and absorbance measurements were immediately collected from 430-460 nm using 1 nm steps. For hyperfolder FPs that did not display a single clean peak at ~447 nm during the initial absorbance scan—representing the denatured chromophore—a 10-30 min time-course was conducted using λ_{abs} = 447 nm and 505 nm. To prevent artificial EC inflation, the *maximum* 447 nm absorbance value from the time-course (see **Extended Data Fig. 5**) was chosen for calculation, thereby yielding the *lowest* possible EC value relative to other points in the data set, since absorbance and concentration are inversely proportional when ε is constant (44,000 M^{-1} cm^{-1} for the denatured GFP-type chromophore). Performed this way, the EC values for the slow-denaturing FPs largely fell within the typical EC range for bright GFPs and YFPs[15]. This strategy applies to "fast-denaturing" avFPs just the same, since the first data point at ~447 nm will be the maximum value when the initial 430-460 nm absorbance scan shows that the FP has denatured.

Quantum yield (QY) values were determined as described[23], using a PTI Quantamaster and the same quartz cuvettes from the absorbance and EC measurements. Experimental samples were run in quadruplicate with the following cross-referenced controls for GFPs/YFPs: fluorescein in 0.1 M NaOH (ϕ = 0.925); and Clover (ϕ = 0.76)[16] and eYFP (ϕ = 0.61)[50] in PBS, pH 7.4. For the LSS-GFPs, the following standards were used: quinine sulfate in 0.1 M H_2SO_4 (ϕ = 0.53)[77]; and EBFP2 (ϕ = 0.56)[78] and mT-Sapphire (ϕ = 0.60)[15] in PBS, pH 7.4.

Refolding

Purified protein in TNG buffer (50 mM Tris-HCl, 150 mM NaCl, 10% glycerol, pH 7.5) was diluted into a solution of 7 M GdnHCl prepared in TNG buffer and supplemented with dithiothreitol (DTT), for final concentrations of 1 µM protein, 6.8 M GdnHCl, and 1 mM DTT. The samples in this solution were fully denatured by heating to 98 °C for 10 min. Next, samples were cooled to RT and briefly examined under a 470 nm handheld LED to confirm total loss of fluorescence. Native samples in the same buffer without GdnHCl were prepared in parallel.

The BioTek Synergy H1 microplate reader was configured for rapid fluorescence endpoint scans at $\lambda_{ex}/\lambda_{em}$ = 495/525 nm for 1 hr at 5 s intervals. 20 µL of native and denatured protein at 1 µM concentration was

dispensed into empty wells of a black 96-well optical plate. Refolding was initiated by ejecting 200 µL TNG buffer supplemented with 1 mM DTT directly into the wells without further mixing (protein $C_f = 0.1$ µM), and the kinetic scan was initiated within 10 s. The data were plotted as the value of the refolding sample divided by the native sample containing 0 M GdnHCl for each time point, thereby giving the fraction folded.

Crystallography

Fluorescent proteins were crystallized using the hanging drop vapor diffusion method with Hampton Research VDX 24-well plates. 2 µL protein (20-40 mg/mL) was gently pipetted into 2 µL reservoir solution on 20 mm diameter siliconized cover glass, sealed with grease atop a well containing 0.5 mL reservoir solution, and the plates were stored at 18 °C. In our hands, the most favorable starting point for avFP crystallization performed this way is with solutions of 0.1 M Tris-HCl pH 8.5, 25-30% PEG 3350 or PEG 4000, and 25-100 mM $MgCl_2$. The most effective PEGs in our hands were PEG 3350, PEG 4000, and PEG 8000. Likewise, the most effective salts were $MgCl_2$, Li_2SO_4, and sodium acetate, in descending order. Screening primarily consisted of optimization around the mentioned conditions with 0.1 M Tris-HCl, pH 8.5, and 18 °C storage in the dark.

hfYFP formed large, smooth, fluorescent yellow crystals after 1 wk in a solution of 0.1 M Tris-HCl pH 8.5, 25% PEG 3350, and 0.2 M sodium acetate. The crystals matured for another week before they were looped out, cryoprotected using the same the reservoir solution ("mother liquor") supplemented with 10% ethylene glycol, and flash frozen in liquid nitrogen. mhYFP formed crystals after 1 wk equilibration against reservoir solution consisting of 0.1 M Tris-HCl pH 8.5, 25% PEG 3350, and 25 mM $MgCl_2$. The solution was a good cryo-condition and did not require supplementation. The mhYFP crystals were looped out and gently "washed" in a 2 µL droplet of reservoir solution ("mother liquor") that was pipetted onto cover glass and handled within a humidified environment to minimize evaporation of the droplet, before the crystal was flash frozen in liquid nitrogen. FOLD6 crystallized after 1 month in 0.1 M Tris-HCl pH 8.5, 30% PEG 4000, and 100 mM $MgCl_2$. The crystals began to emerge from phase-separated green protein globules that coalesced and gradually transitioned from a semi-solid state into the final crystal form over a period of 1 month, after which they were looped out and frozen.

Note that we are treating mfoxYtiPLM as "mhYFP" in this section, although technically its V206K mutant is "mhYFP" (**Supplementary Table 1**). We're indicating this here for accuracy and completeness, although previous studies have shown no significant structural differences from common position 206 substitutions apart from side chain identity (i.e. Ala, Lys, Val)[19,79].

Crystallographic data collection, processing, and refinement

Crystallographic diffraction data were collected at GM/CA CAT 23-IDD of the Advanced Photon Source at Argonne National Laboratory with monochromatic x-rays of 1.033 angstroms at 100 K on a Dectris Pilatus3 6M HPC detector (Dectris Ltd., Switzerland) and processed with XDS[80]. Structural homology model of each respective proteins was generated from the crystal structure of Clover (PDB: 5WJ2)[23] using the online server Swiss-Model[81]. These homology models were used as a molecular replacement search model and yielded a solution with the program Phaser[82]. The initial molecular replacement solution was subjected to multiple rounds of maximum likelihood restrained refinement using PHENIX[83] and manual rebuilding with Coot[84]. Complete data collection and refinement statistics are provided in **Supplementary Table 3**.

Mammalian cell culture and confocal imaging

Low passage HEK293T (ATCC, CRL-3216), HeLa (ATCC, CCL-2), and BE(2)-M17 cells (ATCC, CRL-2267) were routinely cultured in OptiMEM (Gibco) supplemented with 5% fetal bovine serum (VWR) and penicillin-streptomycin (Gibco).

For localization experiments and OSER assay, HeLa cells were passaged into 35 mm culture plates containing a 20 mm glass bottom (MatTek #P35G-1.5-20-C, "MatTek plates") and grown for at least 24 hr before transfection using Turbofect (Thermo Fisher) and 0.5-1.0 µg plasmid. hfYFP folds very quickly, and some organelles naturally require the unfolded polypeptide to pass through a translocon for successful import before folding and expression in the target structure[40,85]. Therefore, new users of hfYFP/mhYFP—or any fast-folding FP—should test several DNA concentrations to ensure proper targeting.

For the OSER assay, live cells were imaged 12-18 hr after transfection using a Zeiss LSM 880 laser-scanning confocal microscope equipped with computer-controlled Zeiss Enhanced Navigation (ZEN) software; an argon-ion laser for with MBS 488 and MBS 458/514 beam splitters for GFP and YFPs, respectively; Plan-Apochromat 10×/0.45 WD=2.0 M27, Plan-Apochromat 40×/1.3 Oil DIC UVVIS-IR, and Plan-Apochromat 63×/1.40 Oil DIC f/ ELYRA objectives; high-sensitivity GaAsP photodetector; configuration for bidirectional scanning and 12-bit image acquisition. FP fusion localization images were acquired at 2048x2048 px resolution with 4 times averaging and represent a single Z-section at 1 Airy unit. OSER assay images were tile scans (4x4) acquired at 1024x1024 px resolution with 4 times averaging using the 40× oil immersion objective. OSER images were stitched using ZEN software and analyzed using the established scoring criteria[14].

To generate representative images of LifeAct-mhYFP following protein-retention expansion microscopy (proExM), HeLa cells were grown on Geltrex-coated 35 mm MatTek dishes with 20 mm glass bottom and transfected using LifeAct-mhYFP ~24 hr after plating. The next day, representative live cells were imaged in phenol red-free culture medium at 63× magnification. The cells were fixed using 4% PFA, taken through the standard proExM protocol as described[86], partially expanded using PBS, pH 7.4, and imaged using the described Zeiss LSM 880. At the time of this experiment, the confocal microscope's 514 nm line was not operational, so the 488 nm argon-ion line was used instead to image mhYFP for the representative proExM images.

Fluorescence retention, proExM

Quantification of fluorescence retention for individual FPs after proExM was conducted in a similar manner as the FP screening method from Tillberg et al.[3], with stock reagents and buffers prepared as described.

Note: in our opinion, the following process for comparing fluorescence retention between FPs after proExM is effective, but we caution that it is highly labor-intensive due to the extreme fragility of the 16-chamber glass slides, the number of replicates necessary (at least 2 hydrogels per FP from separate preps), and the time required to properly transfer and position the hydrogels, image them, and then analyze the large tile scans. Sophisticated registration and unwarping software would facilitate the analysis. Subjectively, we would consider expansion and imaging of mouse coronal sections to be much easier due to morphological landmarks and the relative ease of gelling a tissue section (refer to the coverslip-sandwich strategy in [86]). The 16-chamber format is most useful when comparing many FPs together to address engineering questions, but we recommend using other culture formats for routine proExM, especially 35 mm MatTek dishes with glass-bottom.

To compare the fluorescence retained by various FPs after proExM, 16-chamber No. 1.5 coverglass slides (Grace Bio-Labs, #GBL112358) were coated with Matrigel (Corning, #356235) to improve attachment and morphology on glass. HeLa cells were passaged to reach ~60-80% confluency approximately 24 hr after plating and were transfected using the recommended 96-well format transfection conditions using Turbofect (Thermo Fisher) and 0.2 µg H2B-FP plasmid DNA for nuclear localization. After overnight expression, cells were fixed using room temperature (RT) 4% PFA in PBS, pH 7.4 (Gibco), at RT for 10-15 min. Afterward, the cells were washed for 5 min with 0.1 M glycine in PBS to quench fixation, followed by two washes with PBS for 5 min each. The 16-chamber slide was then imaged using a Keyence BZ-X700 All-in-One Fluorescence Microscope equipped with a PlanFluor DL 4× 0.13/16.50mm PhL air objective, standard GFP filter cube, and computer-controlled motorized stage. After manually focusing each well, 5x5 tile scans of 640x480 px (3x3 binning) resolution were acquired in 8-bit TIFF format under imaging conditions specific to each FP to provide optimal exposure while minimizing oversaturation. The imaging settings for each well were noted.

After acquiring the pre-expansion images of post-fixed cells, samples were treated with acryloyl-X (AcX) (Thermo Fisher, #A20770) in PBS, pH 7.4, overnight at RT while protected from light. The next day, AcX was removed using a micropipette, and samples were washed 3 times for 5 min each using PBS.

The slide was placed on a Kimwipe on a clean benchtop free of dust and debris. Next, the plastic silo chambers that are adhered to the upper silicone spacer were separated from the lower silicone gasket (which itself is adhered to the coverslip surface), by looping dental floss carefully *between the silicone spacer and silicone gasket* from one end of the slide, all the way through and out the other. Alternating between gently pulling from one end of the floss at a time with one hand while delicately holding the slide in place with the other hand (*without* pressing the slide against the benchtop or squeezing it), can be helpful to reduce the risk of coverslip breakage.

After the floss has been passed through, the two gaskets usually remain in their original positions while PBS has seeped between the narrow gaps opened by the dental floss to weaken the adhesive. This way, the chamber and silicone spacer together can be separated from the lower gasket *carefully* using fingertips or forceps to lift the plastic chamber/space gently—beginning from one end of the slide to the other—taking great care to avoid bending the brittle No. 1.5 coverslip. To serve as a reservoir for the gelling step, the lower silicone gasket *must remain attached to the coverslip* cell side up. Since PBS will have leaked out, fresh PBS should be added swiftly to any empty wells to prevent them from drying out (we recommend using a P20 micropipette to dispense the liquid slowly and avoid dislodging cells). Alternatively, Grace Bio-Labs offers a removal tool, but we have had far greater success using dental floss.

The cell side of the coverslip now has only the lower black silicone gasket attached to it. This slide was placed inside a clean, dry, 15 cm culture plate with a 1 µL drop of water beneath it to keep it in place by surface tension. The 15 cm plate provides an enclosure for the slide during gelling and helps transfer it more easily to the incubator. Gelling solution was prepared on ice and distributed into the wells as described after PBS removal[86]. The 15 cm plate lid was placed back on the plate, and the plate was then carried level to a 37 °C dry incubator (normoxic; not a tissue culture incubator) and the gel was polymerized for 60-90 min.

After gelling, the thick parafilm-coated No. 2 slide glass lid that was placed atop the gasket was removed. The black silicone gasket was very carefully and slowly peeled off using blunt forceps, taking the greatest care not to bend the coverslip to any degree. The glass coverslip was then cut using a diamond scribe, with

good practice and eye protection, to separate the coverslip into sections of individual gels, each still attached to a square of glass as described[86]. Thoughtful planning is critical for maintaining sample order when the slide is divided, or else the gels cannot be distinguished.

Digestion buffer was prepared and supplemented with proteinase K (New England Biolabs, #P8107S), dispensed into 12-well plate. The gels were immersed, with their cut glass fragment still attached, into the solution overnight at RT. The next day, the glass was carefully removed with thin forceps as described by Asano et al., with the gels remaining in the 12-well plate[86]. Digestion buffer was removed using a P1000 micropipette or 5 mL serological pipette, taking care not to break the semi-transparent gels. The gels were then washed 3-4 times, ~20 min per wash, using PBS to semi-expand them. An orbital shaker on a very slow setting is optional and can help facilitate diffusion. The PBS-expanded gels were then shrunk back using the same wash step timing, using shrinking solution (1 M NaCl and 60 mM $MgCl_2$ in water) instead of PBS. Gels typically shrink back to ~1.2-1.5x their original size.

The shrunken gels were imaged that same day using the same microscope settings that were recorded for each well from the pre-expanded images. When possible, the original orientation was maintained. Gels were imaged in a No. 1 or No. 1.5 thickness empty glass-bottom 6-well plate or in a MatTek 35 mm glass-bottom plate. Coating the glass surfaces beforehand with poly-L-lysine or poly-D-lysine solution greatly helps prevent the gels from drifting. The hydrogels become very sticky when dry and should be kept moist. Gels were flipped so that the cell-side would face down against the glass (determined by focusing). A metal spatula bent at the end was useful for transferring the gels from the 12-well plate into the glass-bottom imaging vessel, with practice.

The pre- and post-expansion images for each FP were stitched using Keyence BZ-X Analyzer software. Post-expansion images of the shrunken gels were registered by unwarping and aligning in Adobe Photoshop to the pre-expansion image manually for each pair using the screen layer option and transform tool until the image borders overlapped and the orientation was matched. The unwarped post-expansion image was exported as the same file type without compression or further modification. Image pairs were quantified in ImageJ by selecting multiple regions of the stitched image using the rectangle selection tool, saving the ROIs, applying them to the second image, and collecting measurements from each ROI. Oversaturated cells and any severely out-of-focus regions were excluded from analysis. The mean fluorescence intensity (MFI) of each ROI after expansion was expressed as a percentage relative to the pre-expansion MFI for that ROI.

Antibody compatibility

Antibody compatibility images were acquired as described[6]. Briefly, HEK293T cells were transfected with pcDNA3.1-FP for cytosolic expression and were fixed with room temperature (RT) 4% PFA before immunostaining using primary antibodies: Gt α GFP polyclonal, Abcam, #ab6673; Gt α GFP polyclonal, Novus, #NB1001770; Ms α GFP monoclonal, Invitrogen, #A-11120). The secondary antibody Dk α Gt IgG Alexa 555 (Invitrogen, #A-21432) was applied, followed by DAPI to label nuclei. Cells were imaged on a Nikon Eclipse 80i microscope after mounting on slides.

Confocal photobleaching

Photobleaching experiments were performed as described[6] using a Zeiss LSM 880 confocal microscope equipped with a Plan-Apochromat 40×/1.3 Oil DIC UVVIS-IR objective. Live HeLa cells in phenol red-free media were transfected with 0.2 µg H2B-FP plasmid DNA using Turbofect (Thermo Fisher). Pinhole was set to 1 Airy unit ("0.9-µm section"), scan time 316 ms, pixel size 0.83 µm, pixel dwell 4.12 µs, 256 x 256-px frames, and 12-bit depth. Laser power was measured at the objective with a Thorlabs PMD100

power meter equipped with S130VC photodetector (Thorlabs) and was initially set to the minimum power necessary to identify suitable regions of the live HeLa cell cultures for bleaching.

To initiate bleaching, power was raised to 147 μW from the blue diode (LSS FPs), or 18.3 μW (GFPs) or 9.8 μW (YFPs) from the argon-ion laser. Time series images were collected in the 490- to 650-nm emission range using ZEN acquisition software (Zeiss). Uniformly fluorescent nuclei were selected for analysis.

Mitotic cells with bright and condensed/punctate nucleoli were excluded. Scaling of the initial emission rate to 1,000 photons s^{-1} $molecule^{-1}$ at t = 0 s was performed as described to produce the photobleaching plots[87].

Cross-excitation assay

Cross-excitation assays were performed by seeding and transfecting HeLa cells on MatTek plates as described using individual plasmids (0.5 μg DNA per plasmid) encoding LifeAct-eGFP and H2B-mT-Sapphire, or LifeAct-mGreenLantern and H2B-LSSmGFP. The next day, cells were imaged on a Keyence BZ-X700 All-in-One Fluorescence Microscope equipped with an S PlanFluor ELWD ADM 40xC 0.60/3.60-2.80mm Ph2 air objective, 470 nm ex 525 nm em GFP filter cube (Keyence), custom 405 nm ex 525 nm em Keyence BZ-X Cube (Chroma Technology Corp), and computer-controlled motorized stage. Images of 1920x1440 px resolution were acquired in 8-bit TIFF format under identical imaging settings for each of the LifeAct and H2B fusion proteins. Images were prepared using ImageJ.

Supplementary References

42. Arpino, J. a J., Rizkallah, P. J. & Jones, D. D. Crystal Structure of Enhanced Green Fluorescent Protein to 1.35 Å Resolution Reveals Alternative Conformations for Glu222. *PLoS One* **7**, e47132 (2012).

43. Budisa, N. *et al.* Probing the role of tryptophans in Aequorea victoria green fluorescent proteins with an expanded genetic code. *Biol. Chem.* **385**, 191–202 (2004).

44. Kawahara-Kobayashi, A., Hitotsuyanagi, M., Amikura, K. & Kiga, D. Experimental Evolution of a Green Fluorescent Protein Composed of 19 Unique Amino Acids without Tryptophan. *Orig. Life Evol. Biosph.* **44**, 75–86 (2014).

45. Wachter, R. M., Elsliger, M., Kallio, K., Hanson, G. T. & Remington, S. J. Structural basis of spectral shifts in the yellow-emission variants of green fluorescent protein. *Structure* **6**, 1267–1277 (1998).

46. Rekas, A., Alattia, J. R., Nagai, T., Miyawaki, A. & Ikura, M. Crystal structure of venus, a yellow fluorescent protein with improved maturation and reduced environmental sensitivity. *J. Biol. Chem.* **277**, 50573–50578 (2002).

47. Griesbeck, O., Baird, G. S., Campbell, R. E., Zacharias, D. a. & Tsien, R. Y. Reducing the environmental sensitivity of yellow fluorescent protein. *J. Biol. Chem.* **276**, 29188–29194 (2001).

48. Ong, W. J.-H. *et al.* Function and structure of GFP-like proteins in the protein data bank. *Mol. Biosyst.* **7**, 984–992 (2011).

49. Burley, S. K. & Petsko, G. A. Weakly Polar Interactions in Proteins. in *Advances in Protein Chemistry* vol. 39 125–186 (1988).

50. Kremers, G. J., Goedhart, J., Van Munster, E. B. & Gadella, T. W. J. Cyan and yellow super fluorescent proteins with improved brightness, protein folding, and FRET Forster radius. *Biochemistry* **45**, 6570–6580 (2006).

51. Zacharias, D. A., Violin, J. D., Newton, A. C. & Tsien, R. Y. Partitioning of lipid-modified monomeric GFPs into membrane microdomains of live cells. *Science (80-.).* **296**, 913–916 (2002).

52. Nagai, T. *et al.* A variant of yellow fluorescent protein with fast and efficient maturation for cell-biological applications. *Nat. Biotechnol.* **20**, 87–90 (2002).

53. Heim, R., Prasher, D. C. & Tsien, R. Y. Wavelength mutations and posttranslational autoxidation of green fluorescent protein. *Proc. Natl. Acad. Sci.* **91**, 12501–12504 (1994).

54. Cubitt, A. B., Woollenweber, L. A. & Heim, R. Understanding Structure-Function Relationships in the Aequorea victoria Green Fluorescent Protein. in *Methods in Cell Biology* vol. 58 19–30 (1999).

55. Teerawanichpan, P., Hoffman, T., Ashe, P., Datla, R. & Selvaraj, G. Investigations of combinations of mutations in the jellyfish green fluorescent protein (GFP) that afford brighter fluorescence, and use of a version (VisGreen) in plant, bacterial, and animal cells. *Biochim. Biophys. Acta - Gen. Subj.* **1770**, 1360–1368 (2007).

56. Reddy, G., Liu, Z. & Thirumalai, D. Denaturant-dependent folding of GFP. *Proc. Natl. Acad. Sci.* **109**, 17832–17838 (2012).

57. Barondeau, D. P., Putnam, C. D., Kassmann, C. J., Tainer, J. a & Getzoff, E. D. Mechanism and energetics of green fluorescent protein chromophore synthesis revealed by trapped intermediate structures. *Proc. Natl. Acad. Sci. U. S. A.* **100**, 12111–12116 (2003).

58. Li, B., Shahid, R., Peshkepija, P. & Zimmer, M. Water diffusion in and out of the β-barrel of GFP and the fast maturing fluorescent protein, TurboGFP. *Chem. Phys.* **392**, 143–148 (2012).

59. Shinobu, A. & Agmon, N. The hole in the barrel: Water exchange at the GFP chromophore. *J. Phys. Chem. B* **119**, 3464–3478 (2015).

60. Palm, G. J. *et al.* The structural basis for spectral variations in green fluorescent protein. *Nat. Struct. Biol.* **4**, 361–365 (1997).

61. Scott, D. J. *et al.* A Novel Ultra-Stable, Monomeric Green Fluorescent Protein for Direct Volumetric Imaging of Whole Organs Using CLARITY. *Sci. Rep.* **8**, 1–15 (2018).

62. Burley, S. K. & Petsko, G. A. Amino-aromatic interactions in proteins. *FEBS Lett.* **203**, 139–143 (1986).

63. Valley, C. C. *et al.* The methionine-aromatic motif plays a unique role in stabilizing protein structure. *J. Biol. Chem.* **287**, 34979–34991 (2012).

64. Zhong, S. *et al.* Seeing the long tail: A novel green fluorescent protein, SiriusGFP, for ultra long timelapse imaging. *J. Neurosci. Methods* **313**, 68–76 (2019).

65. Brakemann, T. *et al.* A reversibly photoswitchable GFP-like protein with fluorescence excitation decoupled from switching. *Nat. Biotechnol.* **29**, 942–947 (2011).

66. Kredel, S. *et al.* Optimized and Far-Red-Emitting Variants of Fluorescent Protein eqFP611. *Chem. Biol.* **15**, 224–233 (2008).

67. Kredel, S. *et al.* mRuby, a bright monomeric red fluorescent protein for labeling of subcellular structures. *PLoS One* **4**, 1–7 (2009).

68. Akerboom, J. *et al.* Genetically encoded calcium indicators for multi-color neural activity imaging and combination with optogenetics. *Front. Mol. Neurosci.* **6**, 2 (2013).

69. Suzuki, T. *et al.* Development of Cysteine-Free Fluorescent Proteins for the Oxidative Environment. *PLoS One* **7**, e37551 (2012).

70. Hanson, G. T. *et al.* Investigating mitochondrial redox potential with redox-sensitive green fluorescent protein indicators. *J. Biol. Chem.* **279**, 13044–53 (2004).

71. Crameri, A., Whitehorn, E. A., Tate, E. & Stemmer, W. P. C. Improved Green Fluorescent Protein by Molecular Evolution Using DNA Shuffling. *Nat. Biotechnol.* **14**, 315–319 (1995).

72. Nguyen, A. W. & Daugherty, P. S. Evolutionary optimization of fluorescent proteins for intracellular FRET. *Nat. Biotechnol.* **23**, 355–360 (2005).

73. Hsu, S. T. D. *et al.* Folding study of venus reveals a strong ion dependence of its yellow fluorescence under mildly acidic conditions. *J. Biol. Chem.* **285**, 4859–4869 (2010).

74. Gibson, D. G. Enzymatic Assembly of Overlapping DNA Fragments. *Methods Enzymol.* **498**, 349–361 (2011).

75. Jacobs, T. M., Yumerefendi, H., Kuhlman, B. & Leaver-Fay, A. SwiftLib: rapid degenerate-codon-library optimization through dynamic programming. *Nucleic Acids Res.* **43**, e34 (2015).

76. Heckman, K. L. & Pease, L. R. Gene splicing and mutagenesis by PCR-driven overlap extension. *Nat. Protoc.* **2**, 924–932 (2007).

77. Adams, M. J., Highfield, J. G. & Kirkbright, G. F. Determination of Absolute Fluorescence Quantum Efficiency of Quinine Bisulfate in Aqueous Medium by Optoacoustic Spectrometry. *Anal. Chem.* **49**, 1850–1852 (1977).

78. Ai, H. W., Shaner, N. C., Cheng, Z., Tsien, R. Y. & Campbell, R. E. Exploration of new chromophore structures leads to the identification of improved blue fluorescent proteins. *Biochemistry* **46**, 5904–5910 (2007).

79. Von Stetten, D., Noirclerc-Savoye, M., Goedhart, J., Gadella, T. W. J. & Royant, A. Structure of a fluorescent protein from Aequorea victoria bearing the obligate-monomer mutation A206K. *Acta Crystallogr. Sect. F Struct. Biol. Cryst. Commun.* **68**, 878–882 (2012).

80. Kabsch, W. Xds. *Acta Crystallogr. Sect. D Biol. Crystallogr.* **66**, 125–132 (2010).

81. Schwede, T., Kopp, J., Guex, N. & Peitsch, M. C. SWISS-MODEL: An automated protein homology-modeling server. *Nucleic Acids Res.* **31**, 3381–3385 (2003).

82. McCoy, A. J. *et al.* Phaser crystallographic software. *J. Appl. Crystallogr.* **40**, 658–674 (2007).

83. Adams, P. D. *et al.* PHENIX: A comprehensive Python-based system for macromolecular structure solution. *Acta Crystallogr. Sect. D Biol. Crystallogr.* **66**, 213–221 (2010).

84. Emsley, P. & Cowtan, K. Coot: Model-building tools for molecular graphics. *Acta Crystallogr. Sect. D Biol. Crystallogr.* **60**, 2126–2132 (2004).

85. Kashiwagi, S. *et al.* Folding latency of fluorescent proteins affects the mitochondrial localization of fusion proteins. *Cell Struct. Funct.* **44**, 183–194 (2019).

86. Asano, S. M. *et al.* Expansion Microscopy: Protocols for Imaging Proteins and RNA in Cells and Tissues. *Curr. Protoc. Cell Biol.* **80**, 1–41 (2018).

87. Shaner, N. C. *et al.* Improving the photostability of bright monomeric orange and red fluorescent proteins. *Nat. Methods* **5**, 545–551 (2008).

Supplementary Figures & Tables

Supplementary Tables & Figures begin on the next page. One Table or Figure is included per page.

Supplementary Tables & Figures begin on the next page. One Table or Figure is included per page.

Supplementary Table 1. Melting temperature and amino acid sequences of mutants generated in this study, relative to Clover.

Clover[a] →	I167	A206	E124	G232	K101	T105	C48	C70	L221	F223	D234	S147	N149	H203	V68	A69	F46	Other	T_m
FOLD1	T	-	-	-	-	-	-	-	-	-	-	-	-	-	-	-	-	-	87.6
mFOLD1	T	**K**	-	-	-	-	-	-	-	-	-	-	-	-	-	-	-	-	N.D.
mF1Y	T	**K**	-	-	-	-	-	-	-	-	-	-	Y	-	-	-	-	-	84.9
mF1VPK	T	**K**	V	-	-	-	-	-	-	-	-	P	K	-	-	-	-	-	N.D.
F2B	T	V	V	D	-	-	-	-	-	-	-	-	-	-	-	-	-	-	N.D.
mF2B	T	V	V	D	-	-	-	-	K	R	-	-	-	-	-	-	-	-	N.D.
mF2BK	T	V	V	D	-	-	-	-	K	R	-	-	K	-	-	-	-	-	87.0
mF2BK-ΔG	T	V	V	D	-	-	-	-	K	R	-	-	K	-	-	-	-	ΔG4	92.8
**mGreenLantern**[b]	T	**K**	V	D	-	-	-	-	K	R	-	-	K	-	-	-	-	-	87.2
mF2BKK (DMN)	T	**K**	V	D	-	-	-	-	K	R	N	-	K	-	-	-	-	-	N.D.
mF2B2	T	V	V	D	-	-	-	-	K	R	N	-	-	-	-	-	-	-	N.D.
F3C	T	V	V	D	E	Y	-	-	-	-	N	-	-	-	-	-	-	-	N.D.
F3CP	T	V	V	D	E	Y	-	-	-	-	N	P	-	-	-	-	-	-	N.D.
mF3CP	T	V	V	D	E	Y	-	-	K	R	-	P	-	-	-	-	-	-	N.D.
mF3CPK	T	V	V	D	E	Y	-	-	K	R	-	P	K	-	-	-	-	-	86.5
Clover-C48S/C70V	-	-	-	-	-	-	S	V	-	-	-	-	-	-	-	-	-	-	76.6[e]
FOLD4	T	V	V	D	E	Y	S	V	-	-	-	-	-	-	-	-	-	-	86.2
mFOLD4	T	V	V	D	E	Y	S	V	K	-	-	-	-	-	-	-	-	-	84.3
mF4Y	T	V	V	D	E	Y	S	V	K	-	-	-	Y	-	-	-	-	-	85.2
mF4Y-SR	T	V	V	D	E	Y	S	V	K	-	-	**R**	-	-	-	-	-	-	84.3
mF4Y-RK	T	**K**	V	D	E	Y	S	V	K	-	-	**R**	-	-	-	-	-	-	84.2
mF4Y-RKH	T	**K**	V	D	E	Y	S	V	K	-	-	**R**	-	-	-	-	-	E222H	85.6[e]
F4P	T	V	V	D	E	Y	S	V	-	-	-	P	-	-	-	-	-	-	87.4
mF4P	T	V	V	D	E	Y	S	V	K	-	-	P	-	-	-	-	-	-	87.3[e]
mF4P-ti	-	V	V	D	E	Y	S	V	K	-	-	P	-	-	-	-	-	-	85.2
FOLD6	T	V	V	D	E	Y	S	V	-	-	-	P	-	-	L	-		-	90.0
FOLD6-HL	T	V	V	D	E	Y	S	V	-	-	-	P	-	-	L	-		H169L	89.8
mfoxY	T	V	V	D	E	Y	S	V	K	R	N	-	-	Y	L	M	L	-	92.8
mfoxYY	T	V	V	D	E	Y	S	V	K	R	N	-	Y	Y	L	M	L	-	N.D.
mFOLD7	T	V	V	D	E	Y	-	-	K	R	N	P	-	Y	L	M	L	-	90.6
**hfYFP**[c]	-	V	V	D	E	Y	S	V	K	R	N	-	-	Y	L	M	L	-	94.2
mfoxY-Y203F	-	V	V	D	E	Y	S	V	K	R	N	-	-	**F**	L	M	L	-	94.5
mhYFP-K	-	**K**	V	D	E	Y	S	V	K	R	N	-	-	Y	L	M	L	-	94.0
mfoxYtiPLM	-	V	V	D	E	Y	S	V	K	R	N	P	-	Y	L	M	L	L195M	93.6
**mhYFP**[d]	-	**K**	V	D	E	Y	S	V	K	R	N	P	-	Y	L	M	L	L195M	92.8
mfoxYtiPMKH	-	**K**	V	D	E	Y	S	V	K	R	N	P	-	Y	L	M	L	L195M E222H	88.9
mfoxYStr	T	V	V	D	E	Y	S	V	K	R	N	-	-	Y	L	M	L	YF[f]	83.5
mfoxYStr-K	T	**K**	V	D	E	Y	S	V	K	R	N	-	-	Y	L	M	L	YF[f]	84.1
mfoxYY3Str-K	-	**K**	V	D	E	Y	S	V	K	R	N	-	Y	Y	L	M	L	YF[f]	N.D.
mfoxYY3Str	-	V	V	D	E	Y	S	V	K	R	N	-	Y	Y	L	M	L	YF[f]	81.6
mfoxYY3Str-SR-ti	-	V	V	D	E	Y	S	V	K	R	N	**R**	Y	Y	L	M	L	YF[f]	80.7

[a] All FPs listed (except Clover and Clover-C48S/C70V) additionally carry the F64L/S72A/S175G mutations. Amino acid abbreviations that might be difficult to distinguish visually are bolded for clarification. Note: all mutants appearing in this list that are listed *above* the “Clover-C48S/C70V” row were previously reported in Campbell et al., *PNAS*, 2020, and are included here for continuity and comparison.

[b] mGreenLantern (mGL) = mF2BK-K (DMD).

[c] hfYFP = mfoxYti.

[d] mhYFP = hfYFP-S147P/L195M = mfoxYtiPLM-V206K = mfoxYtiPMK.

[e] FPs that showed more than one melt peak are indicated. The secondary peak for Clover-C48S/C70V, mF4P, and mF4Y-RKH, appear at 88.9 °C, 96.7 °C, and 76.8 °C, respectively. See Fig. 2g and Supplementary Fig. 5c-f for melt curves.

[f] YF: F145Y/N146F.

N.D.: not determined.

Supplementary Table 2. Quantification of chemical and thermal denaturation experiments from Fig. 2.

**Protein**	T_m (°C)	87 °C persistence $t_{½}$ (min)	Kinetic unfolding, GdnHCl $t_{½}$ (min)	Equilibrium unfolding, GdnHCl $C_{½}$ (M)	Kinetic unfolding, GdnSCN $t_{½}$ (min)	Equilibrium unfolding, GdnSCN $C_{½}$ (M)	NaOH persistence, $t_{½}$ (s)
hfYFP	94.2	40.2	∞	[c]∞	9.3	3.2	18
mhYFP[c]	92.8	N.D.	N.D.	N.D.	6.4	3.0	120
FOLD6	90.0	N.D.	N.D.	N.D.	N.D.	N.D.	142
mF4P	87.3	[b]3.1	N.D.	N.D.	0.1	2.2	84
mGreenLantern	87.2	17.4	206.1	5.5	1.6	2.3	0
Superfolder GFP	86.4	1.5	2.9	4.3	0	0.1	0
eGFP	80.3	0	<1	4.0	0	0.2	0
mClover3	80.1	0	1.8	3.8	0	0.1	0
eYFP	72.9	0	2.4	1.0	0	0.1	1
mNeonGreen	68.0	0	9.2	2.0	0	0.8	0

[a] T_m data here are from Extended Data Table 1 for the purpose of comparison (typical experimental error: ± 2 °C).

∞ Hyperfolder YFP was stable in GdnHCl for at least 3 months at room temperature in 7 M GdnHCl, pH 7.4.

[b] mF4P shows a double-exponential melting process with a fast phase $t_{½}$ = 3.1 min and τ = 4.5 min during which 80% of the initial fluorescence is lost. This is followed by an extended slow phase (τ = 122 min) of $t_{½}$ = 85 min, eventually intercepting hyperfolder YFP's mono-exponential curve at t = 200 min and continuing with it for the rest of the process.

[c] mhYFP is hfYFP-S147P/L195M/V206K.

[c] Fluorescence at the highest GdnHCl concentration of 6.3 M was ~50% greater than the initial value in buffer without GdnHCl.

Supplementary Table 3. Crystallographic data collection and refinement statistics.

Protein	**hfYFP**	**mhYFP**	**FOLD6**
Resolution range	27.39 - 1.74 (1.802 - **1.74**)	27.28 - 1.63 (1.688 - **1.63**)	29.63 - 1.21 (1.253 - **1.21**)
Space group	$C222_1$	$C222_1$	$P6_4$
Unit cell	51.994 66.872 143.308 90 90 90	52.347 66.659 142.465 90 90 90	61.438 61.438 112.38 90 90 120
Total reflections	78920 (8237)	198902 (19649)	306013 (2199)
Unique reflections	25423 (2564)	31489 (3070)	64116 (1788)
Multiplicity	3.1 (3.2)	6.3 (6.3)	4.8 (1.2)
Completeness (%)	97.44 (99.77)	97.66 (99.19)	87.89 (24.56)
Mean I/sigma(I)	8.58 (2.46)	25.79 (4.36)	18.36 (0.36)
Wilson B-factor	29.43	24.84	16.31
R-merge	0.08541 (0.3248)	0.03641 (0.3444)	0.03744 (1.128)
R-meas	0.1034 (0.3919)	0.03978 (0.3754)	0.04124 (1.552)
R-pim	0.05718 (0.2156)	0.01581 (0.1481)	0.017 (1.06)
CC1/2	0.989 (0.873)	0.999 (0.942)	1 (0.29)
CC*	0.997 (0.966)	1 (0.985)	1 (0.671)
Reflections used in refinement	25423 (2564)	30785 (3070)	64115 (1789)
Reflections used for R-free	1333 (134)	1144 (114)	2006 (59)
R-work	0.2015 (0.3351)	0.1503 (0.1923)	0.1458 (0.3245)
R-free	0.2344 (0.3473)	0.1875 (0.2104)	0.1710 (0.3412)
CC(work)	0.955 (0.806)	0.968 (0.899)	0.975 (0.468)
CC(free)	0.943 (0.716)	0.948 (0.847)	0.959 (0.433)
Number of non-hydrogen atoms	2016	2061	2200
macromolecules	1789	1836	1903
ligands	55	19	19
solvent	172	206	278
Protein residues	227	227	229
RMS(bonds)	0.01	0.015	0.004
RMS(angles)	1.38	1.83	0.99
Ramachandran favored (%)	97.75	98.65	98.66
Ramachandran allowed (%)	2.25	1.35	1.34
Ramachandran outliers (%)	0	0	0
Rotamer outliers (%)	0	0.99	1.87
Clashscore	3.28	4.62	2.86
Average B-factor	33.67	32.9	23.11
macromolecules	32.95	32.01	21.51
ligands	37.34	21.17	14.82
solvent	39.97	41.92	34.61
Number of TLS groups		1	

[a] Statistics for the highest-resolution shell are shown in parentheses.

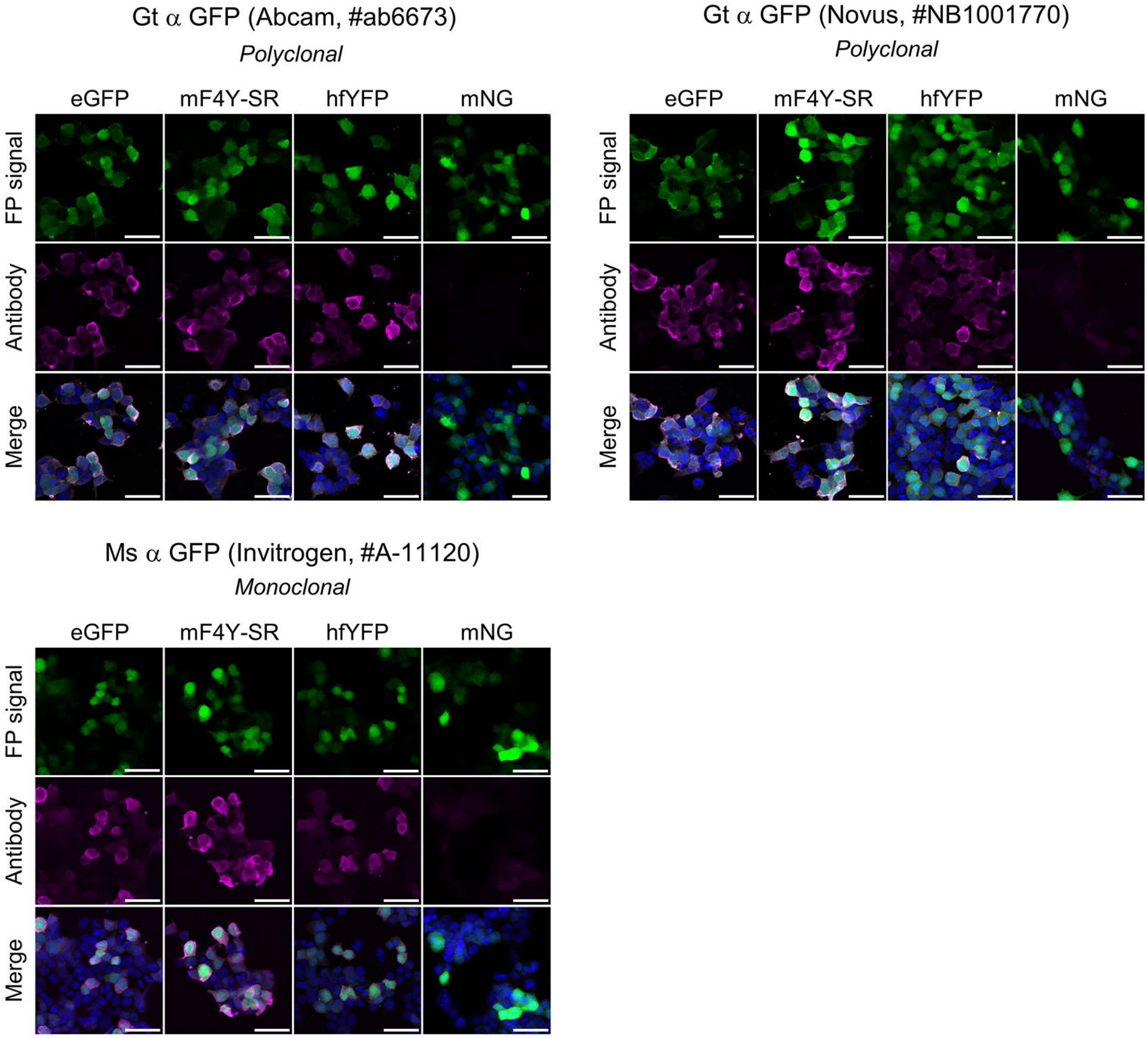

Supplementary Figure 1. mhYFP is compatible with commercially available antibodies designed for eGFP. Antibody signal co-localized with HEK293T cells expressing cytosolic FPs. mF4Y-SR is a hyperfolder mutant related to mGreenLantern (mutations are listed in Supplementary Table 1). mNeonGreen is derived from from *B. lanceolatum* rather than *A. victoria* and therefore serves as a negative control. Antibody channel: Alexa 555 conjugated Donkey secondary antibody. Merged image features DAPI in the blue channel. mGreenLantern was shown to react with the same set of antibodies used here in Campbell et al., *PNAS*, 2020. Images represent a single experiment. Scale bars, 25 μm.

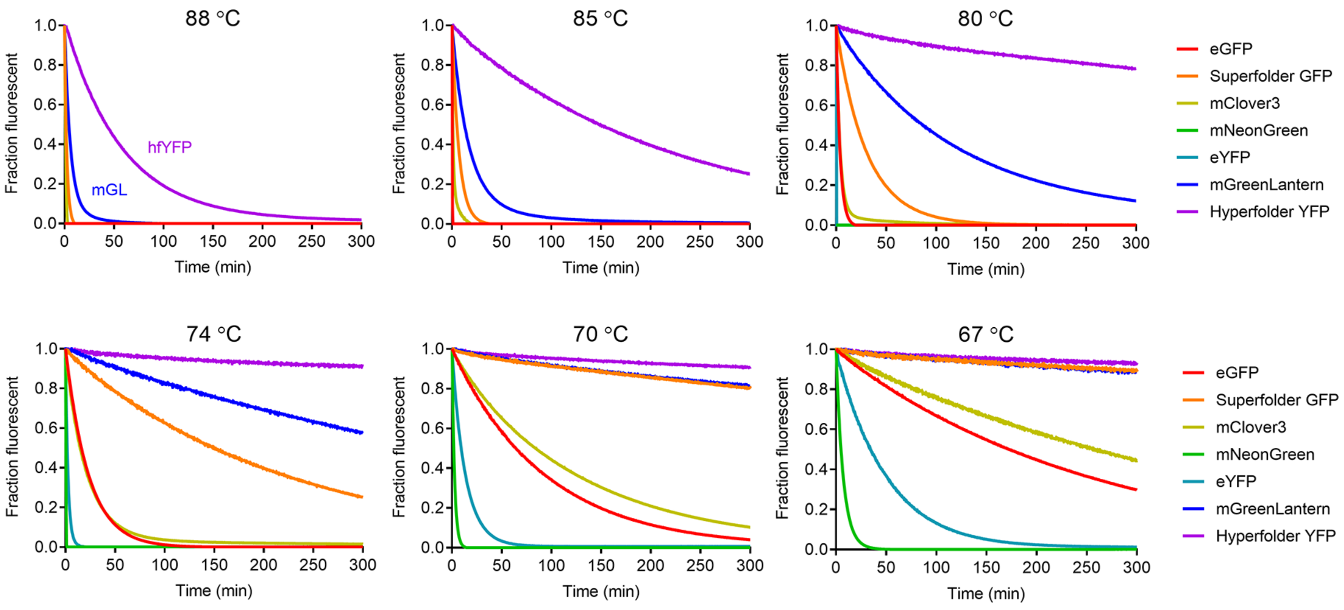

Supplementary Figure 2. Isothermal melting of fluorescent proteins. FPs were rapidly heated to the target temperatures in separate wells using the gradient function of a real-time PCR machine. Fluorescence intensity was quantified every 30 s using the FAM filter. Data are plotted by normalizing the intensity values to the first data point at t = 0 min. Values from the same data set at t = 60 min were used to construct the summary panel in **Fig. 2f**.

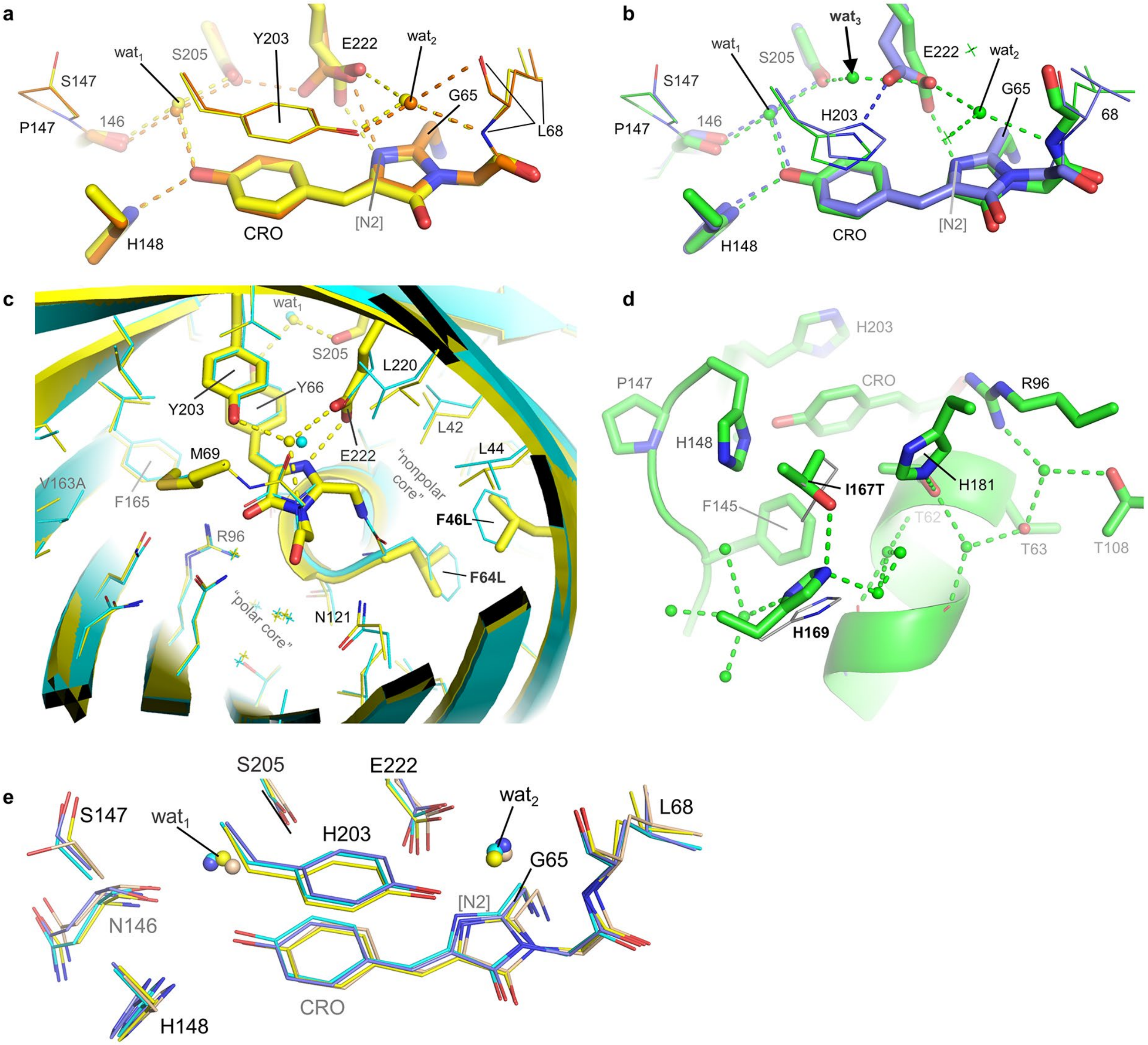

Supplementary Figure 3. Chromophore environments of hyperfolder proteins. (**a**) Superposition of hfYFP (yellow) and mhYFP (orange) crystal structures. mhYFP displays an atypical E222 conformation for a YFP: E222 is hydrogen-bonded (H-bonded) to [N2] and S205 instead of [N2] and wat$_2$. [N2]: nitrogen atom of the chromophore imidazolinone ring. (**b**) FOLD6 (green) and Clover (blue) superposition. In Clover, E222 is H-bonded to [N2] but not to S205, and this is the only E222 conformation detected. The FOLD6 E222 is H-bonded to [N2] and to wat$_3$, completing a proton wire to the chromophore phenolate. Wat$_1$ and wat$_2$ are highly conserved structural water molecules in avFPs, whereas wat$_3$ is rarely observed. (**c**) Overhead view of hfYFP (yellow) and Citrine (cyan) crystal structures. The chromophore phenolate points toward β-strand 7 at the 12 o'clock position. The polar and nonpolar cores of the protein are indicated at the 7 o'clock and 3 o'clock positions. The F46L and F64L mutations in hfYFP, relative to Citrine, are located at the 3 o'clock position (bolded). The hfYFP H-bond network, identical to Citrine's, is shown. (**d**) FOLD6 (green) cutaway side view of the protein's polar core, with the central α-helix visible and the chromophore phenolate pointing toward β-strand 7. H203 is stacked on top. sfGFP side chains of H169 and I167 are visible in grey line form for comparison. Green dashes: H-bonds. Crosses: various water molecules not directly involved in the proton wire. (**e**) Chromophore conservation in avFPs shown by superposition of hfYFP (yellow), eYFP (blue), Citrine (cyan), and Venus (pink). These four FPs share the same general chromophore H-bond network and side-chain orientations (± ~0.5 Å). The same structural water molecules observed in most avFPs, which we refer to as wat$_1$ and wat$_2$, are observed here. hfYFP (PDB: 7UGR), mhYFP (PDB: 7UGS), FOLD6 (PDB: 7UGT), Citrine (PDB: 1HUY), Venus (PDB: 1MYW).

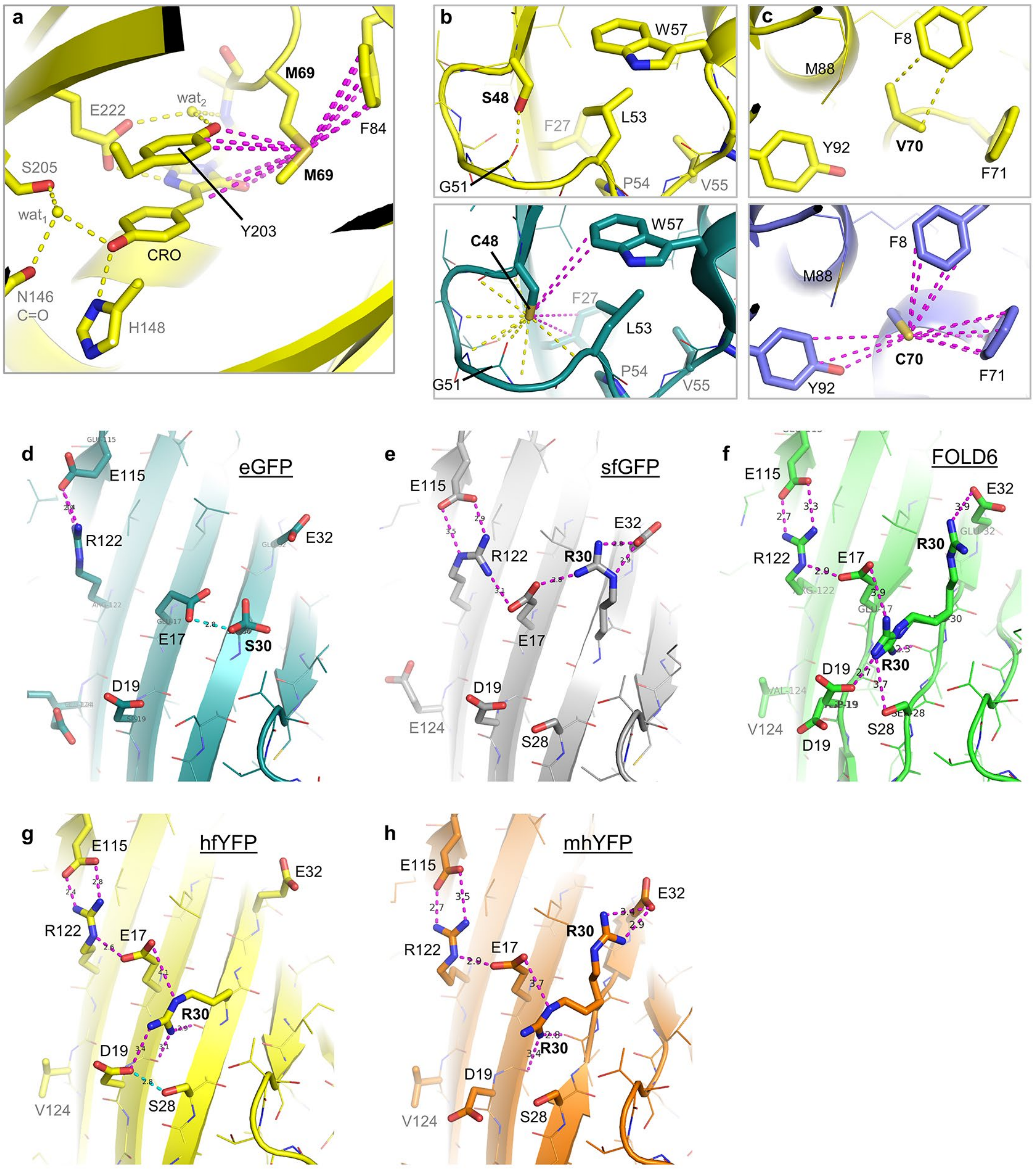

Supplementary Figure 4. Proposed role of C48/C70 and surface hydrogen bonds in avFPs. (**a**) Structure of the hfYFP (yellow) chromophore environment as observed from β-strand 8. Y203 is pictured above the chromophore. Yellow dashes: hfYFP H-bond network. Magenta dashes: sulfur-aromatic interactions from M69 with interaction distances averaging ~6 Å—these are expected to stabilize the chromophore (CRO), Y203, and especially the F84 side chain to which the sulfur atom shows an orientational preference. The configuration improves hydrophobic packing relative to the wild-type Q69 residue. (**b**) The C48S mutation in hfYFP (yellow, top image) produces a tight 2.3 Å H-bond between the S48 hydroxyl side chain and the G51 carbonyl. L53 has rotated relative to its conformation in eGFP (turquoise, bottom image) to stabilize W57 through vdW forces. In eGFP, C48 is approximately 5.5 Å from the electropositive edges of the F27 and W57 rings. The foreground D216 side-chain in these images has been omitted to improve visualization of residue 48, but note that W57 $N_{\varepsilon 1}$ is H-bonded to D216 $O_{\delta 1}$ in every structure. (**c**) The C70V mutation in hfYFP (yellow, top image) eliminates the lone-pair electrons of the C70 sulfur atom that in eYFP (purple, bottom image) and other avFPs, can interact with the electropositive edges of the F8, F71, and Y92 ring to stabilize them (magenta dashes: sulfur-aromatic interactions). These sulfur-aromatic interactions likely provide greater support than vdW forces alone. Consequently, ring positions have shifted 0.3-0.5 Å in hfYFP relative to Venus to adjust to new vdW distances (yellow dashes) from V70. (**d**) Example of stabilizing salt bridges in avFPs. FPs were aligned in PyMOL and are each presented in the same orientation. Magenta dashed lines: salt bridges of <4 Å. Cyan dashed lines: H-bond of ≤ 3.0 Å. eGFP displays an H-bond between E17 and S30 in addition to the E115-R122 salt bridge observed in each of the depicted structures. (**e**) sfGFP. (**f**) FOLD6, with multiple conformations observed for the critical R30 superfolder mutation that stabilizes several neighboring side-chains. (**g**) In hfYFP, R30 is found in a single conformation that secures it with four ionic bonds, one each to the E17 and D19 carboxylates, and two to main-chain carbonyls. (**h**) mhYFP shows a sfGFP-like salt bridge network with an additional R30 conformation observed. hfYFP (PDB: 7UGR), eYFP (PDB: 1YFP), Citrine (PDB: 1HUY), Venus (PDB: 1MYW).

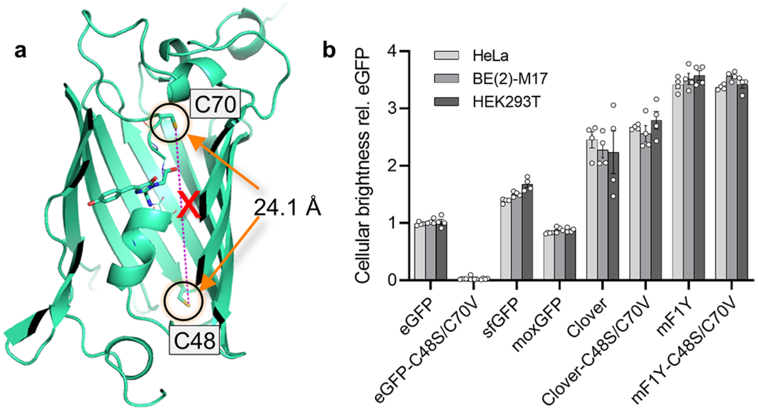

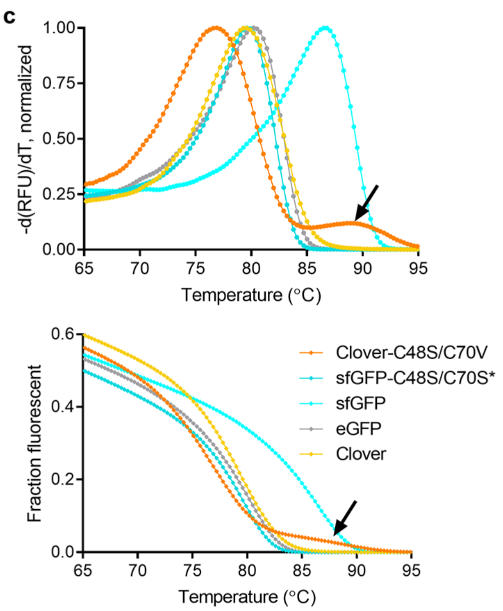

d

Protein*	λ_{ex}	λ_{em}	ϕ	ε ($M^{-1} cm^{-1}$)	Mol. bright	Cell. bright	pK_a	T_m (°C)	Maturation (min)
eGFP	488	507	0.71	53,400	38	1.0	6.0	80.3	28
eGFP-C48S/C70V	488	510	0.67	48,200	32	0.02	6.0	N.D.	N.D.
sfGFP	485	508	0.67	51,300	34	1.7	6.2	86.4	37
sfGFP-C48S/C70S*	487	509	0.58†	52,500	30†	0.9	6.1	79.5	50
Clover	505	515	0.75	96,600	72	2.2	6.5	79.5	15
Clover-C48S/C70V	506	516	0.75	99,700	75	2.8	6.5	76.6	31

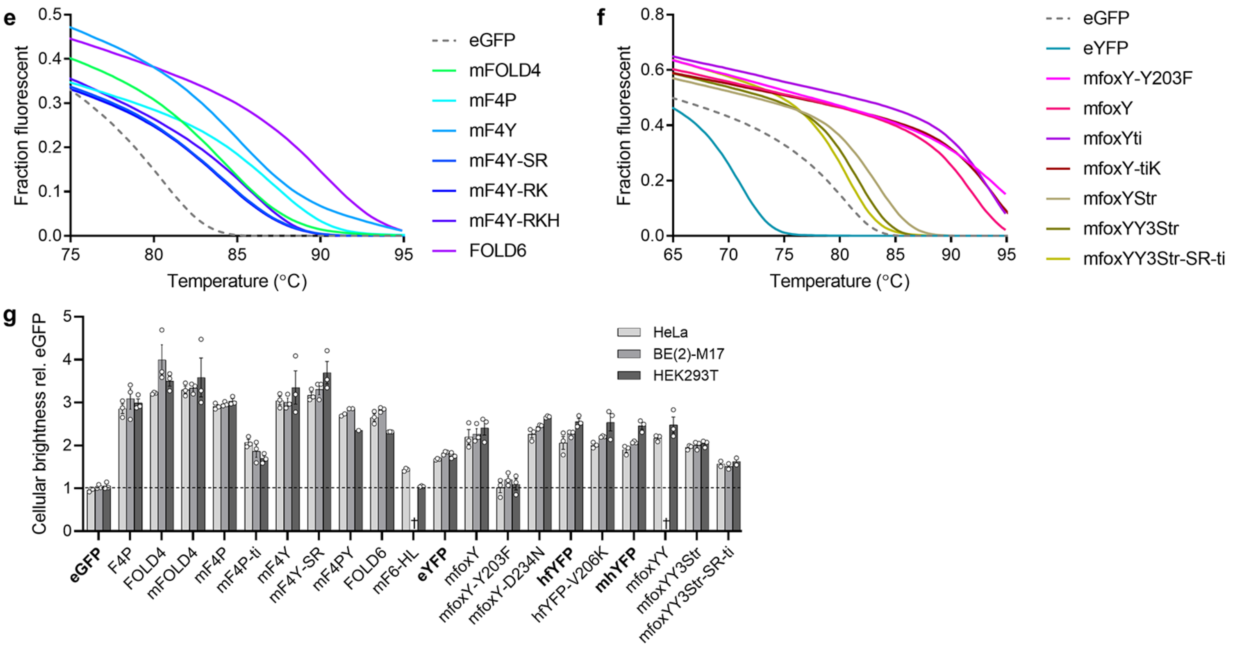

Supplementary Figure 5. Characterization of cysteine-free mutants and selected library members. (**a**) Crystal structure of Clover (PDB: 5WJ2), with cysteines indicated. In all avFPs, C48 and C70 are situated ~24 Å apart (magenta dotted line) and cannot form a disulfide bond under native conditions in the properly folded protein. (**b**) Comparison of cellular brightness values between “wild-type” FPs and cysteine-free variants (Methods). Mean ± s.e.m., n = 4 experimental replicates, each averaging 3 transfections. (**c**) Top: fluorescence of FPs during a 0.3 °C/min temperature ramp from 25-100 °C using a real-time PCR machine with FAM filter. Data are normalized to the fluorescence intensity value at 25 °C. The black arrow indicates a prolonged secondary melting phase in Clover-C48S/C70V that was not observed in the other FPs. The $T_m = x$ when $y = 1$. Asterisk symbol: sfGFP-C48S/C70S is moxGFP. Bottom: data from the same experiment plotted as the negative first derivative of the change in fluorescence, normalized to max for each FP (arbitrary units). (**d**) Spectroscopic characterization of cysteine-free mutants and their brightness in HEK293T cells. The same data for eGFP, sfGFP, and Clover are shown in Extended Data Table 1. Asterisk symbol: human codon optimized sfGFP-C48S/C70S is “moxGFP,” from Costantini et al., *Nat. Comm*., 2015. Dagger symbol: the quantum yield (ϕ) value is cited from Costantini et al.; however, we recommend treating that value as an estimate, since their methods for QY determination were not reported. All other data in the table were produced in our lab in this study. (**e**) Melting curves for a subset of C48S/C70V green and (**f**), yellow fluorescent mutants compared to eGFP and eYFP. (**g**) Cellular brightness for each FP in three mammalian cell lines (Methods). Dagger symbol: mF6-HL and mfoxYY were not tested in BE(2)-M17 cells. Mean ± s.e.m., n = 3 replicate experiments, each averaging 4 independent transfections.

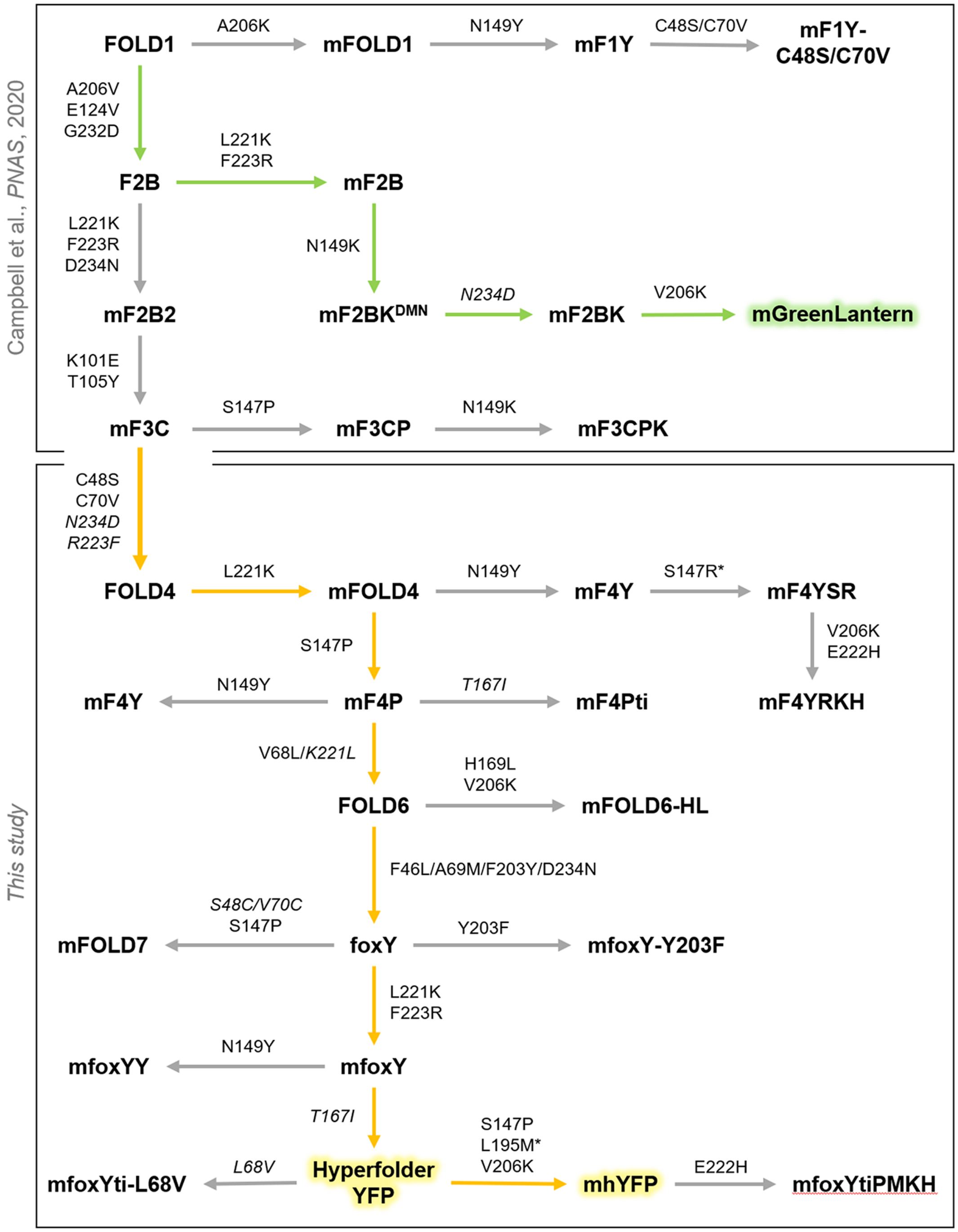

Supplementary Figure 6. Mutation map of spectroscopically characterized hyperfolder GFP/YFP mutants. Green and yellow arrows indicate the cumulative mutations and steps leading to mGreenLantern and the final hyperfolder YFP (hfYFP) variants, respectively. *Italicized* mutations signify reversion at that site to the "wild-type" Clover amino acid identity. *S147R and L195M are *de novo* mutations originating in this study from PCR errors that occurred during site-directed mutagenesis. The S147R mutation was independently reported by another research group for a different purpose during the preparation of this manuscript (Zhong et al., *J. Neurosci. Met.*, 2019).

```
               1         10        20        30        40        50
               |         |         |         |         |         |
mGL            MVSKGEELFTGVVPILVELDGDVNGHKFSVRGEGEGDATNGKLTLKFICTTGKLPVPWPT
hfYFP          MVSKGEELFTGVVPILVELDGDVNGHKFSVRGEGEGDATNGKLTLKLISTTGKLPVPWPT
mhYFP          MVSKGEELFTGVVPILVELDGDVNGHKFSVRGEGEGDATNGKLTLKLISTTGKLPVPWPT
LSSA12         MVSKGEELFTGVVPILVELDGDVNGHKFSVRGEGEGDATNGKLTLKLISTTGKLPVPWPT
LSSmGFP        MVSKGEELFTGVVPILVELDGDVNGHKFSVRGEGEGDATNGKLSLKLISTTGKLPVPWPT

               60        70        80        90        100       110
               |         |         |         |         |         |
mGL            LVTTLGYGVACFARYPDHMKQHDFFKSAMPEGYVQERTISFKDDGTYKTRAEVKFEGDTL
hfYFP          LVTTLGYGLMVFARYPDHMKQHDFFKSAMPEGYVQERTISFEDDGYYKTRAEVKFEGDTL
mhYFP          LVTTLGYGLMVFARYPDHMKQHDFFKSAMPEGYVQERTISFEDDGYYKTRAEVKFEGDTL
LSSA12         LVTTLSYGLMVFARYPDHMKQHDFFKSAMPEGYVQERTISFEDDGYYKTRAEVKFEGDTL
LSSmGFP        LVTTLSYGQMVFARYPDNMKQHDFFKSAMPEGYVQERTISFEDDGYYKTRAEVKFEGDTL

               120       130       140       150       160       170
               |         |         |         |         |         |
mGL            VNRIVLKGIDFKEDGNILGHKLEYNFNSHKVYITADKQKNGIKANFKTRHNVEDGGVQLA
hfYFP          VNRIVLKGIDFKEDGNILGHKLEYNFNSHNVYITADKQKNGIKANFKIRHNVEDGGVQLA
mhYFP          VNRIVLKGIDFKEDGNILGHKLEYNFNPHNVYITADKQKNGIKANFKIRHNVEDGGVQLA
LSSA12         VNRIVLKGIDFKEDGNILGHKLEYNFNSHNVYITADKQKNGIKANFKIRHNVEDGGVQLA
LSSmGFP        VNRIVLKGIDFKEDGNILGHNLEYNFNSHNVYITADKQKNGIKANFKIRHNVEDGGVQLA

               180       190       200       210       220       230
               |         |         |         |         |         |
mGL            DHYQQNTPIGDGPVLLPDNHYLSHQSKLSKDPNEKRDHMVLKERVTAAGITHDMDELYK*
hfYFP          DHYQQNTPIGDGPVLLPDNHYLSYQSVLSKDPNEKRDHMVLKERVTAAGITHDMNELYK*
mhYFP          DHYQQNTPIGDGPVLMPDNHYLSYQSKLSKDPNEKRDHMVLKERVTAAGITHDMNELYK*
LSSA12         DHYQQNTPIGDGPVLLPDNHYLSIESVLSKDPNEKRDHMVLKDFVTAAGITHDMNELYK*
LSSmGFP        DHYQQNTPIGDGPVLLPDNHYLSIQSKLSKDPNEKRDHMVLKERVTAAGITHDMNELYK*
```

Note: FP engineers traditionally append the C-terminal amino acids "…GMDELYK*" (asterisk for stop codon) to most FPs, if they are not already present. The FPs shown here instead use C-terminal sequence "…**D**MDELYK*" (G232D mutation) for mGL, and "…**D**M**N**ELYK*" (G232D/D234N mutations) for hfYFP, mhYFP, LSSA12, and LSSmGFP. These mutations are important for the stability properties and brightness of these proteins. For optimal performance (and properties as characterized in this manuscript), we recommend preserving the complete C-terminal sequences.

Supplementary Figure 7. Fluorescent protein amino acid sequence comparison to mGreenLantern. Mutations that are most important to the properties of the specific FP are highlighted. The alignment is bolded when a residue has changed in any FP relative to mGL at the indicated amino acid position.

4% PFA
(Fig. 3a)

FP	Mean (%)	± s.d. (%)	Cells
eGFP	84.8	29.6	556
mClover3	82.9	29.0	484
mNG	42.5	9.3	450
mGL	74.9	20.9	329
hfYFP	81.3	18.6	227
eYFP	60.7	19.8	329

Tukey's multiple comparisons test	Summary	Adjusted P Value
eGFP vs. mClo3	ns	0.7533
eGFP vs. mNG	****	<0.0001
eGFP vs. mGL	****	<0.0001
eGFP vs. hfYFP	ns	0.3783
eGFP vs. eYFP	****	<0.0001
mClo3 vs. mNG	****	<0.0001
mClo3 vs. mGL	****	<0.0001
mClo3 vs. hfYFP	ns	0.9578
mClo3 vs. eYFP	****	<0.0001
mNG vs. mGL	****	<0.0001
mNG vs. hfYFP	****	<0.0001
mNG vs. eYFP	****	<0.0001
mGL vs. hfYFP	*	0.0188
mGL vs. eYFP	****	<0.0001
hfYFP vs. eYFP	****	<0.0001

4% PFA + 5% Glut
(Fig. 3b)

FP	Mean (%)	± s.d. (%)	Cells
eGFP	65.7	24.3	506
mClover3	64.1	18.4	506
mNG	29.3	6.7	504
mGL	65.7	15.4	378
hfYFP	75.2	17.7	236
eYFP	55.0	11.1	304

Tukey's multiple comparisons test	Summary	Adjusted P Value
eGFP vs. mClo3	ns	0.7121
eGFP vs. mNG	****	<0.0001
eGFP vs. mGL	ns	>0.9999
eGFP vs. hfYFP	****	<0.0001
eGFP vs. eYFP	****	<0.0001
mClo3 vs. mNG	****	<0.0001
mClo3 vs. mGL	ns	0.7341
mClo3 vs. hfYFP	****	<0.0001
mClo3 vs. eYFP	****	<0.0001
mNG vs. mGL	****	<0.0001
mNG vs. hfYFP	****	<0.0001
mNG vs. eYFP	****	<0.0001
mGL vs. hfYFP	****	<0.0001
mGL vs. eYFP	****	<0.0001
hfYFP vs. eYFP	****	<0.0001

proExM
(Fig. 3e)

FP	Mean (%)	± s.d. (%)	ROIs
eGFP	50.4	18.1	50
mClover3	50.1	11.8	21
mNG	56.3	23.8	33
mGL	63.5	19.7	27
hfYFP	66.3	19.3	38
eYFP	N.D.	N.D.	N.D.

Dunnett's multiple comparisons test	Summary	Adjusted P Value
eGFP vs. mClo3	ns	>0.9999
eGFP vs. mNG	ns	0.4905
eGFP vs. mGL	*	0.0189
eGFP vs. hfYFP	***	0.0007

4% PFA
(Extended Data Fig. 7e)

FP	Mean (%)	± s.d. (%)	Cells
LSSA12	74.6	26.5	1747
LSSmGFP	71.6	28.0	1740
eGFP	68.6	26.2	1758
mT-Sapphire	62.0	32.8	1715
mAmetrine	40.7	22.6	1765

Tukey's multiple comparisons test	Summary	Adjusted P Value
mT-Sapphire vs. mAmetrine	****	<0.0001
mT-Sapphire vs. LSSA12	****	<0.0001
mT-Sapphire vs. LSSmGFP	****	<0.0001
mT-Sapphire vs. eGFP	****	<0.0001
mAmetrine vs. LSSA12	****	<0.0001
mAmetrine vs. LSSmGFP	****	<0.0001
mAmetrine vs. eGFP	****	<0.0001
LSSA12 vs. LSSmGFP	*	0.0103
LSSA12 vs. eGFP	****	<0.0001
LSSmGFP vs. eGFP	*	0.0105

4% PFA + 5% Glut
(Extended Data Fig. 7f)

FP	Mean (%)	± s.d. (%)	Cells
LSSA12	55.1	25.6	1034
LSSmGFP	68.9	13.2	1035
eGFP	37.4	19.3	1035
mT-Sapphire	53.4	26.0	1031
mAmetrine	24.6	16.8	1034

Tukey's multiple comparisons test	Summary	Adjusted P Value
mT-Sapphire vs. mAmetrine	****	<0.0001
mT-Sapphire vs. LSSA12	ns	0.4069
mT-Sapphire vs. LSSmGFP	****	<0.0001
mT-Sapphire vs. eGFP	****	<0.0001
mAmetrine vs. LSSA12	****	<0.0001
mAmetrine vs. LSSmGFP	****	<0.0001
mAmetrine vs. eGFP	****	<0.0001
LSSA12 vs. LSSmGFP	****	<0.0001
LSSA12 vs. eGFP	****	<0.0001
LSSmGFP vs. eGFP	****	<0.0001

Supplementary Figure 8. Statistics tables. Complete statistics for **Fig. 3a-b**, **Fig. 3e**, and **Extended Data Fig. 7e-f**. Data were analyzed using one-way ANOVA with Tukey's multiple comparisons test. Please refer to the tables for p values. Full details of these experiments are available in Methods.

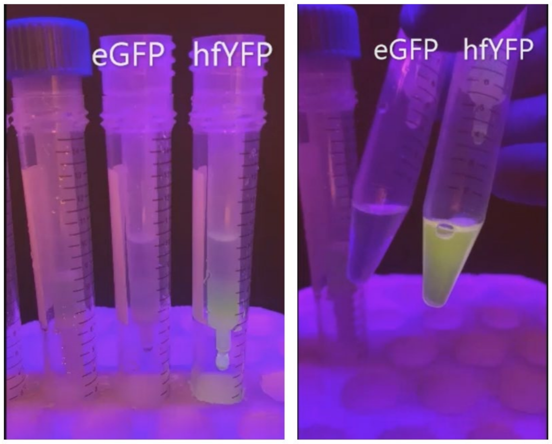

Supplementary Video 1. Fluorescence-assisted elution of streptavidin fusion proteins under denaturing conditions. Intact fusions of LSSmGFP (left), eGFP (center), or hfYFP (right) with streptavidin (SAV) as depicted in Fig. 5g are shown. Fusion proteins were obtained from solubilized inclusion bodies (IBs). As described in Methods, Denaturing Purification buffer (20 mM phosphate, 300 mM NaCl, 6 M GdnHCl, pH 7.4) containing 250 mM imidazole was added to Ni-NTA columns to elute the His_6-tagged fusion proteins. Elution was monitored by eye at the benchtop using 470 nm illumination for eGFP and hfYFP and orange filter glasses. LSSmGFP is not excited by 470 nm light (see Supplementary Video 2) for elution of LSSmGFP-SAV fusion protein under 405 nm illumination). eGFP was immediately denatured by the GdnHCl at the inclusion body (IB) solubilization stage (see Fig. 5f) and is therefore nonfluorescent, whereas hfYFP remains fluorescent throughout the entire process. Video is a 2-frames-per-second time lapse.

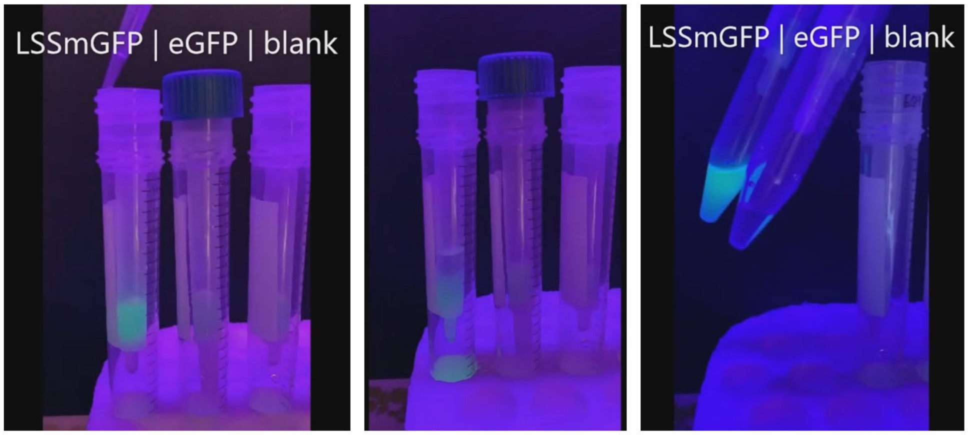

Supplementary Video 2. Fluorescence-assisted elution of streptavidin fusion proteins under denaturing conditions. Time-lapse video at 2 frames per second showing elution of LSSmGFP-SAV fusion protein under 405 nm illumination (no emission filter). Denaturing Purification Buffer with 250 mM imidazole (solution contains 6 M GdnHCl) is added to the column to elute the protein. Intact fusions of LSSmGFP (left), eGFP (center), or hfYFP (right) with streptavidin (SAV) as depicted in Fig. 5g are shown. Refer to figure legend for Supplementary Video 1 for additional details. hfYFP is not excited by 405 nm light, and although eGFP would be weakly excited, it was denatured by GdnHCl. Therefore, only LSSmGFP is fluorescent here under 405 nm illumination.
