# Supplementary material for: Chemically stable fluorescent proteins for advanced microscopy
Source: Nat Methods. 2022 Nov 7;19(12):1612–21. doi: 10.1038/s41592-022-01660-7 (PMC9718679; doi:10.1038/s41592-022-01660-7)
Supplement: Supplementary file 2 — Reporting Summary [file 41592_2022_1660_MOESM2_ESM.pdf]

Corresponding author(s): Benjamin C. Campbell, Ph.D.

Last updated by author(s): September 4, 2022

Reporting Summary

Nature Research wishes to improve the reproducibility of the work that we publish. This form provides structure for consistency and transparency in reporting. For further information on Nature Research policies, see our Editorial Policies and the Editorial Policy Checklist.

Statistics

For all statistical analyses, confirm that the following items are present in the figure legend, table legend, main text, or Methods section.

n/a	Confirmed	
☐	☒	The exact sample size (*n*) for each experimental group/condition, given as a discrete number and unit of measurement
☐	☒	A statement on whether measurements were taken from distinct samples or whether the same sample was measured repeatedly
☐	☒	The statistical test(s) used AND whether they are one- or two-sided<br>*Only common tests should be described solely by name; describe more complex techniques in the Methods section.*
☒	☐	A description of all covariates tested
☒	☐	A description of any assumptions or corrections, such as tests of normality and adjustment for multiple comparisons
☐	☒	A full description of the statistical parameters including central tendency (e.g. means) or other basic estimates (e.g. regression coefficient) AND variation (e.g. standard deviation) or associated estimates of uncertainty (e.g. confidence intervals)
☐	☒	For null hypothesis testing, the test statistic (e.g. *F*, *t*, *r*) with confidence intervals, effect sizes, degrees of freedom and *P* value noted<br>*Give P values as exact values whenever suitable.*
☒	☐	For Bayesian analysis, information on the choice of priors and Markov chain Monte Carlo settings
☒	☐	For hierarchical and complex designs, identification of the appropriate level for tests and full reporting of outcomes
☒	☐	Estimates of effect sizes (e.g. Cohen's *d*, Pearson's *r*), indicating how they were calculated

Our web collection on statistics for biologists contains articles on many of the points above.

Software and code

Policy information about availability of computer code

Data collection	Biotek Gen5 software (v2.09.2), BZ-X Analyzer (v1.4.0.1), ZEN microsocopy software (latest version available at the time; late 2019).
Data analysis	GraphPad Prism 8, Microsoft Excel, XDS, Phaser, PHENIX, Coot

For manuscripts utilizing custom algorithms or software that are central to the research but not yet described in published literature, software must be made available to editors and reviewers. We strongly encourage code deposition in a community repository (e.g. GitHub). See the Nature Research guidelines for submitting code & software for further information.

Data

Policy information about availability of data

All manuscripts must include a data availability statement. This statement should provide the following information, where applicable:

- Accession codes, unique identifiers, or web links for publicly available datasets
- A list of figures that have associated raw data
- A description of any restrictions on data availability

Crystal structures have been uploaded to the PDB under accession codes 7UGR (hfYFP), 7UGS (mhYFP), and 7UGT (FOLD6). Plasmids have been deposited at Addgene. DNA sequences of the fluorescent proteins have been uploaded to GenBank: OP373686 (hfYFP), OP373687 (mhYFP), OP373688 (LSSA12), OP373689 (LSSmGFP). Additional data that support the findings of this study are available from the corresponding author upon reasonable request.

Field-specific reporting

Please select the one below that is the best fit for your research. If you are not sure, read the appropriate sections before making your selection.

☒ Life sciences ☐ Behavioural & social sciences ☐ Ecological, evolutionary & environmental sciences

For a reference copy of the document with all sections, see nature.com/documents/nr-reporting-summary-flat.pdf

Life sciences study design

All studies must disclose on these points even when the disclosure is negative.

Sample size	Biochemical and spectroscopic assays involving purified protein were highly reproducible, repeated >=2-3 times. Technical replicates were used within replicate experiments, and sample sizes and methodology are indicated. For the experiment involving fluorescence retention after fixation, whole tile scans of chamber slides were used for quantification. For the CLEM experiment, multiple random FOVs were used for scoring. Cellular assays were repeated on different days and the data were averaged as indicated. In some figures, error bars are omitted for clarity, such as when thousands of data points are present from kinetic measurements. Number of experimental replicates are listed in the figure legends. Sample sizes are comparable to field standard assays from the most recent several years for cell-based characterization experiments. We concluded that one experiment (glyoxal fixation) showed over-expression artifacts, and a decision was made during revision to remove the experiment, it was not repeated.
Data exclusions	Data were not excluded.
Replication	We have found the experiments to be reproducible. Experiments were typcially repeated several times with consistent results.
Randomization	No experimental groups, so no need for randomization.
Blinding	No experimental groups, so no need for blinding.

Reporting for specific materials, systems and methods

We require information from authors about some types of materials, experimental systems and methods used in many studies. Here, indicate whether each material, system or method listed is relevant to your study. If you are not sure if a list item applies to your research, read the appropriate section before selecting a response.

Materials & experimental systems

n/a	Involved in the study	
☐	☒	Antibodies
☐	☒	Eukaryotic cell lines
☒	☐	Palaeontology and archaeology
☒	☐	Animals and other organisms
☒	☐	Human research participants
☒	☐	Clinical data
☒	☐	Dual use research of concern

Methods

n/a	Involved in the study	
☒	☐	ChIP-seq
☒	☐	Flow cytometry
☒	☐	MRI-based neuroimaging

Antibodies

Antibodies used	Gt a GFP (Abcam, #ab6673); Gt a GFP (Novus, #NB1001770); Ms a GFP (Invitrogen, #A-11120)
Validation	These are commonly used, highly validated commercial antibodies.

Eukaryotic cell lines

Policy information about cell lines

Cell line source(s)	HEK293T (ATCC, CRL-3216), HeLa (ATCC, CCL-2), and BE(2)-M17 cells (ATCC, CRL-2267)
Authentication	Provided by ATCC and authenticated by morphology comparison to ATCC sample images.
Mycoplasma contamination	Cells were determined mycoplasma negative using a commercial fluorescence kit.
Commonly misidentified lines (See ICLAC register)	To our knowledge and from ICLAC, these cell lines are not commonly misidentified.
